# Preclinical Evaluation of a Radiolabeled Anti-PSMA Dimeric Aptamer in a Murine Model of Human Prostate Cancer

**DOI:** 10.3390/molecules31030493

**Published:** 2026-01-31

**Authors:** Akesh Sinha, Darpan N. Pandya, Prabhakar Eeka, Olcay Boyacioglu, William H. Gmeiner, Thaddeus J. Wadas

**Affiliations:** 1 Department of Radiology, University of Iowa, Iowa City, IA 52242, USA; akesh-sinha@uiowa.edu (A.S.); darpan-pandya@uiowa.edu (D.N.P.); prabhakar-eeka@uiowa.edu (P.E.); 2 Department of Food Engineering, Adnan Menderes University, Zafer 09100, Turkey; oboyaci@adu.edu.tr; 3 Department of Cancer Biology, Wake Forest University Health Sciences, Winston-Salem, NC 27109, USA; bgmeiner@wakehealth.edu

**Keywords:** zirconium-89, aptamer, radiochemistry, prostate cancer, prostate-specific membrane antigen

## Abstract

Prostate cancer is the third-leading cause of cancer death in men. Prostate-specific membrane antigen (PSMA) is a robust biomarker that is expressed in approximately 80% of patients diagnosed with prostate cancer; several theranostic strategies have emerged based upon targeting this biomarker. This report describes a dimeric aptamer complex (DAC) which is selective for PSMA^+^ cancer cells and is amenable to derivatization with additional diagnostic and therapeutic molecules. Confocal microscopy confirmed the selective nature of the DAC for PSMA^+^ LNCAP tumor cells. In addition, the affinity of the DAC for the PSMA protein was determined to be 2.16 ± 0.15 nM using biolayer interferometry (BLI). In proof-of-principle studies, this DAC was biotinylated (BioDAC; A10), complexed with streptavidin (SA), and radiolabeled with the positron-emitting radioisotope zirconium-89 (^89^Zr: t_½_ = 78.4 h, β^+^: 22.8%) to form the radiopharmaceutical [^89^Zr]Zr-Df-SA-BioDAC ([^89^Zr]Zr-A12). Acute biodistribution studies revealed elevated levels of radioactivity in PSMA^+^ tumors when compared to PSMA^−^ tumors. Radioactivity retention in the kidney was high due to the presence of streptavidin, while radioactivity retention in the liver was comparable with that of other radiolabeled aptamer complexes. Accordingly, the data suggests that the radiopharmaceutical will need to be redesigned using a strategy that is not reliant on a biotin–streptavidin paradigm before additional preclinical assessments are made and clinical translation can be attempted.

## 1. Introduction

Prostate cancer (PCa) is a complex disease with multiple risk factors including advanced age, genetic predisposition, genetic alteration and racial disparities [1]. It primarily originates in the luminal epithelia cells of the prostate gland, is the third-leading cause of cancer death in men, and despite advances in its detection and treatment, it remains a significant cause of cancer-related deaths globally [1,2]. One reason for this alarming trend is the inability to effectively diagnose and treat PCa-induced metastasis, which causes most of the morbidity and mortality among patients. Once metastasis has occurred, the 5-year survival rate is only 30% [3].

Over the past few decades, the diagnosis of prostate cancer has evolved. While digital rectal exams, hematological surveillance for increased prostate specific antigen secretion or transrectal- or transperitoneal-guided ultrasound with biopsy are used clinically, their limitations have been well documented in the literature [1,4]. The utilization of clinical imaging techniques such as magnetic resonance imaging (MRI), computed tomography (CT), single photon emission computed tomography (SPECT) and positron emission tomography (PET) have all been explored to improve the way PCa is diagnosed, staged, and treated. Clinical MRI and CT are imaging modalities that provide anatomical information. Currently, the former is utilized for diagnosis, surveillance and staging while the latter is used primarily for disease staging. SPECT is a nuclear medicine technique that requires the injection of a radiopharmaceutical, which emits gamma ray photons, and it is considered the current standard for PCa staging [4]. The role of clinical PET/CT continues to evolve in the management of PCa; this is primarily due to advances in radiopharmaceutical development and scanner design [5,6,7,8]. Like SPECT, PET is a nuclear medicine technique that requires the use of a radiopharmaceutical containing a positron-emitting radioisotope, but when compared to SPECT, PET demonstrates greater advantages with respect to sensitivity and resolution. When combined with CT or MRI, superior anatomical resolution can be combined with PET data that reflects pathophysiology, receptor expression levels, enzyme activity and cellular metabolism [9,10,11,12,13].

Due to positron annihilation and coincidence detection, PET/CT has demonstrated superior resolution and quantitation compared to clinical SPECT/CT imaging, and this has led to intense radiopharmaceutical development for PCa imaging and management in the clinic [14,15,16,17,18,19,20,21,22,23,24,25,26,27,28,29,30,31]. One of the most promising biomarkers to be identified is the dimeric transmembrane protein prostate specific membrane antigen (PSMA) [32]. Although PSMA is expressed in the endothelial cells of various malignancies, it has been found to be overexpressed on the apical plasma membrane of PCa cells at all stages of disease, including poorly differentiated metastatic mCRPC carcinomas [33]. This expression pattern has fostered intense research around developing radiopharmaceuticals for the imaging and therapy of PCa; it has led to the FDA approval of several agents including [^18^F]F-DCFPyL, [^68^Ga]Ga-PSMA-11 and [^177^Lu]Lu-PSMA-617 [30,34,35,36,37,38,39,40,41,42,43,44,45,46,47,48].

Aptamers are a class of molecules composed of single-stranded RNA (ssRNA) or DNA (ssDNA) oligonucleotides with the ability to fold into three-dimensional structures and bind to their target in a similar manner to their antibody protein counterparts [49,50,51]. Typically, aptamers are generated by “Systematic Evolution of Ligands by Exponential Enrichment” (SELEX) [52,53,54], which is a process involving the iterative incubation, isolation, elution and amplification of a randomized oligonucleotide library to a target biomolecule. In theory, this process produces aptamers with high selectivity and specificity to the target biomolecule of interest. Aptamers demonstrate unique advantages when compared to monoclonal antibodies (mAbs), including economical production, high target affinity, low immunogenicity, improved tumor accessibility, improved pharmacokinetics and chemistries that facilitate the conjugation of radionuclides or anti-cancer drugs [49,51,55]. Given their unique properties, several research groups have developed aptamers that are selective for the PSMA protein. For example, RNA aptamer A-10 and its truncated variants were investigated for their ability to target PSMA [52,55,56,57,58]. However, its ability to internalize within PCa cells was limited [52]. Boyacioglu et al. identified a dimeric DNA aptamer complex (DAC) worthy of further study since it was selectively internalized by PSMA^+^ cells at elevated levels [59]. In that work, the research team demonstrated through cell studies involving PSMA^+^ C4-2 and PSMA^−^ PC-3 cells that the DAC could be loaded successfully with doxorubicin (DOX). Additionally, they demonstrated that DOX could be selectively delivered to C4-2 cells where it underwent a slow endosomal release and nuclear localization, which resulted in enhanced cytotoxicity. Contrastingly, the DOX-loaded DAC displayed a minimal cytotoxic effect in PC-3 cells. In this study, we sought to develop a PET radiopharmaceutical that could monitor DOX delivery in a non-invasive way. As a first step in this process, we chose to extend the observations of Boyacioglu et al. by reconfirming the aptamer’s ability to bind PSMA on cells, evaluating the affinity of the DAC for PSMA using biolayer interferometry (BLI), radiolabeling this molecule with ^89^Zr (^89^Zr: t_½_ = 78.4 h, β^+^: 22.8%) and evaluating the pharmacokinetics of the resulting radiopharmaceutical in acute biodistribution studies in a preclinical mouse model of human PSMA^+^ prostate cancer.

## 2. Results and Discussion

### 2.1. Synthesis and Quality Control of Aptamer Complexes

The membrane-bound glycoprotein PSMA has emerged as an important biomarker in the development of diagnostic and therapeutic radiopharmaceuticals for prostate cancer since it demonstrates up to a 1000-fold greater expression on the epithelium of prostate adenocarcinoma and positively correlates with several clinical indicators of aggressiveness and poor outcomes [60]. In this work we sought to develop a PET radiopharmaceutical for the imaging of PSMA^+^ PCa that was based upon the dimeric aptamer complex A3 (Table 1), which was originally discovered by Boyacioglu and colleagues [59]. We focused on this design since previous research demonstrated that it was selectively internalized by PSMA^+^ cells, could function as a high-capacity chemotherapy delivery system, and contained design elements to improve stability in vivo. Based upon the structure of A3, several other dimeric aptamer complexes were designed to probe PSMA targeting using in vitro cell studies and in vivo biodistribution experiments. The dimeric aptamer complexes and their derivatives that were utilized in these studies are described in Table 1. Unless otherwise specified, the aptamer number is used as the unique molecule identifier throughout the remainder of the text.

While aptamers A1–A9 were purchased directly from Aptagen, Inc. and used without further modification, aptamer A10 was produced by annealing aptamers A2 and A9, which was biotinylated at the 5′ end to form the biotinylated dimeric DNA aptamer. Incorporation of biotin at the 5′ end provided a convenient tag that would allow us to exploit the SA–biotin interaction to study A10’s affinity for PSMA using BLI. It also provided a convenient strategy to rapidly generate a radiopharmaceutical (vide infra).

The SYBR-stained native gel demonstrated that A10 had migrated more slowly and demonstrated a distinct electrophoretic pattern when compared to the monomeric DNA aptamer strands, A2 and A9. Although A10 represented the major reaction product, its purity was estimated to be no greater than 60% on the native PAGE gel (Figure S1). Thus, A10 was further purified using gel elution and SE-HPLC to increase its purity to 95% (Figures S1 and S2).

MALDI-MS analysis of the A11 bioconjugate revealed an observed mass of 15.72 kDa. This corresponds to approximately 0.36 Df molecules conjugated to the monomeric SA protein, which has a mass of 15.45 kDa (Figure S3). MALDI-MS of A10 and A12 failed to generate sufficient signal for accurate analysis. Thus, we applied mass photometry (MP) to study the purity and molecular mass of A10 and A12. MP is a label-free optical method for the solution-based measurements of biomolecules [61]. The measurement principle relies on quantifying the change in surface reflectivity because of individual biomolecules and their complexes, which is in proportion to their molecular mass. This technique has been used to characterize a variety of biomolecules including DNA. MP of A10 revealed an average mass of 45.5 ± 6.5 kDa (Figure S4). MP of the A12 system revealed an average mass of 58 ± 11 kDa (Figure S5).

### 2.2. Confocal Microscopy Analysis and Affinity Constant Determination

Although previously established by Boyacioglu et al., confocal microscopy was conducted with the Cy3-Cy5-labeled dimeric aptamer complex A6 to reconfirm its selectivity for PSMA^+^ cells. Both the nucleus and the cell membrane were fluorescently labeled using DAPI and WGA-Alexa 488, respectively, so that the location of A6 could be easily established in relation to these cellular structures (Figure S6). Confocal microscopy images demonstrate internalization of A6 within PSMA^+^ LNCaP cells. However, A6 was not observed to localize within the nucleus. Contrarily, negative control aptamer A7 did not demonstrate association with the same cell system (Figure S6). To further corroborate selectivity, A6 was incubated with PSMA^−^ PC-3 cells under similar conditions. However, binding on the cell membrane or internalization was not observed during these experiments (Figure S6). This result reconfirmed those by Boyacioglu and colleagues and demonstrated that the bioconjugation of a reporter molecule did not alter the characteristics of the native dimeric aptamer complex [59].

To further evaluate the in vitro properties of the dimeric aptamer complex, we performed BLI. BLI is a label-free optical technique for real-time analysis of interacting biomolecules and is commonly used for measuring reaction rates and dissociation constants of biomolecular interactions [62,63,64]. We chose this route rather than conventional binding studies using the analogous radiopharmaceutical as a consequence of the small quantities of dimeric aptamer complex that could be generated at any one time during the course of our experiments. To accomplish this, A1 was modified with a biotin moiety at the 5′ end of the oligonucleotide strand to form A9 and then annealed with A2 to form the dimeric aptamer complex A10, which was immobilized on a streptavidin-coated biosensor (Figure S7). Luckily, this modification did not interfere with the annealing process required to form A10, and binding was measured in the presence of different concentrations of human PSMA as an analyte at the limiting biotinylated A10 concentration. Using a 1:1 binding model, the dissociation constant (K_d_) describing the interaction between A10 and human PSMA was observed to be 2.16 ± 0.15 nM (Figure S7). This is consistent with other PSMA-targeting ligands such as the J591 mAb (1.83 nM [65]) and anti-PSMA aptamers such as A10-3 (5 nM [59]), A10-3.2 (2.9 nM [56]) and A9g (5 nM [56]).

### 2.3. Radiochemical Synthesis and in Vitro Serum Stability of [^89^Zr]Zr-A11 and [^89^Zr]Zr-A12

Our initial attempts to develop a radiopharmaceutical based upon a dimeric aptamer complex that had the chelator desferrioxamine (Df) directly attached to the DNA strands resulted in a low yield of the Df-based bioconjugate. When used in radiochemical studies we observed extremely low radiochemical yields and radiochemical purities that were deemed insufficient for studies in vivo. While not fully explored, we surmised that this was most likely the result of the low number of Df molecules attached to the DNA strands and the probability of the chelator being inaccessible once the bioconjugate adopted its preferred conformation in solution during radiochemical synthesis.

**Figure 1:**
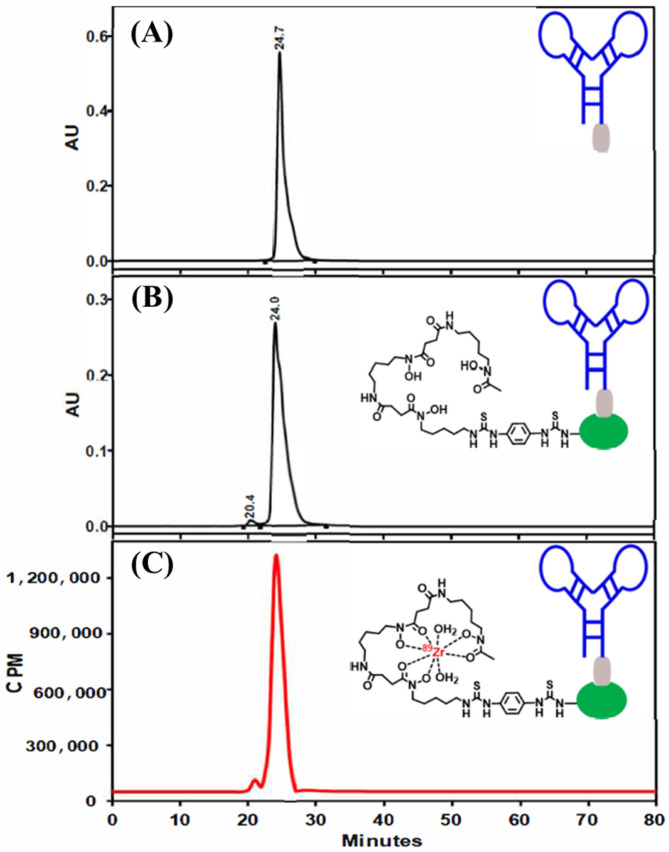
Quality control of [^89^Zr]Zr-A12. (**A**) UV-HPLC chromatogram (280 nm) of A10. (**B**) UV-HPLC chromatogram (280 nm) of A12. (**C**) Radio-HPLC chromatogram of [^89^Zr]Zr-A12. Chromatograms demonstrate that the radiopharmaceutical can be prepared with high purity.

**Scheme 1:**
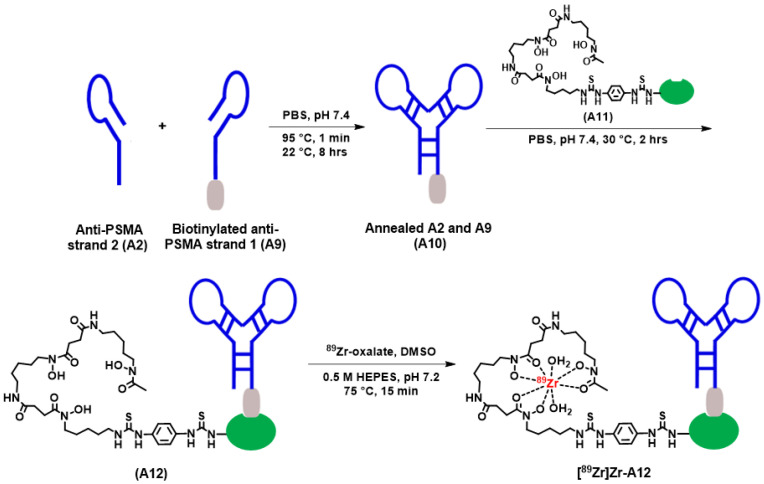
Modified preparative route used to prepare the biotinylated dimer (A10) and conjugate (A12) and subsequent radiolabeling with zirconium-89.

To circumvent these low yields, we adopted a three-step approach to achieve our goal of a radiolabeled dimeric aptamer complex. This approach, which is depicted in Scheme 1, included (1) the annealing of aptamers A2 and A9 to form A10, (2) the conjugation of A11 with A10 to form A12 (Figure 1 and Figure S8), and (3) the radiolabeling of A12 with ^89^Zr. While we acknowledge this strategy added additional steps to the radiochemistry process, it did provide a straightforward radiochemistry solution that allowed us to generate a radiopharmaceutical that could be carried forward for in vivo studies. Furthermore, although other radioisotopes are available for radiochemical studies [66], we chose to use ^89^Zr given our prior experience with this radionuclide [67,68,69,70,71,72,73,74,75], its expanding application beyond antibodies and nanoparticles [76,77,78,79,80], and because it would allow us to track the distribution and excretion of the radiopharmaceutical over time. Specifically, using our previously published methods, [^89^Zr]Zr-A12 was synthesized using [^89^Zr]Zr-oxalate in the presence of n-acetyl-L-cysteine (NAC) as a radioprotectant, which was used to reduce the formation of free radicals that might be detrimental to the radiopharmaceutical product [67]. The radiochemical purity was determined to be greater than 95%, while the radiochemical yield was observed to be 97% (Figure 1, Figure S9 and Figure S10). The specific activity (A_s_; MBq µg^−1^) was determined to be 0.13 ± 0.006 (n = 5). Once prepared, an aliquot of [^89^Zr]Zr-A12 was incubated with human serum for seven days to determine its resistance to degradation. The results of those studies are shown in Table 2 and Figure S11. By the end of the seven-day study, [^89^Zr]Zr-A12 was observed to be 95% intact, suggesting the resulting radiopharmaceutical was resistant to ^89^Zr transchelation by serum proteins over the time course of this experiment.

**Scheme 2:**
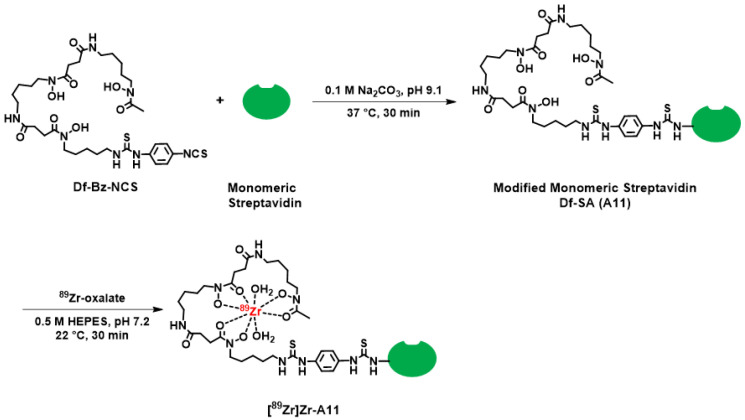
Modified preparative route used for monovalent streptavidin (SA) modification with Df-Bz-NCS and subsequent labeling with zirconium-89 to form [^89^Zr]Zr-A11.

Realizing that [^89^Zr]Zr-A12 is a multi-component system consisting of a dimeric aptamer complex, biotin and SA, we prepared [^89^Zr]Zr-A11 (Scheme 2) to compare its in vitro stability and in vivo biodistribution with those of [^89^Zr]Zr-A12 in order to estimate the influence of monovalent SA on the stability and pharmacokinetics of [^89^Zr]Zr-A12. Similar to the synthesis of [^89^Zr]Zr-A12, preparation of [^89^Zr]Zr-A11 proceeded using our modified conditions that required the reaction of A11 with [^89^Zr]Zr-oxalate in the presence of NAC [67]. The radiolabeling yield (%) was determined to be greater than 96%, while the radiochemical purity (%) exceeded 97% (Figures S12 and S13). The specific activity (A_s_; MBq µg^−1^) was observed to be 0.15 ± 0.005 (*n* = 5). Once prepared, an aliquot of [^89^Zr]Zr-A11 was incubated with human serum for seven days to determine its resistance to degradation. The results of those studies are shown in Table 2. By the end of the seven-day study, [^89^Zr]Zr-A11 was observed to be 97% intact, suggesting minimal transchelation of ^89^Zr by serum proteins over time (Figure S14).

### 2.4. Biodistribution Studies in Tumor-Bearing Mice

Biological clearance and tissue retention properties of [^89^Zr]Zr-A11 and [^89^Zr]Zr-A12 were investigated through biodistribution studies using a murine xenotransplantation model consisting of nude mice bearing LNCaP and PC-3 tumors in contralateral flanks. We chose the LNCaP and PC-3 cell lines for the xenograft model since the former cell line exhibits high expression of membrane-bound PSMA, while the latter cell line is devoid of PSMA expression and would allow us to monitor the radiopharmaceutical’s selectivity for PSMA in vivo [81,82,83,84].

**Figure 2:**
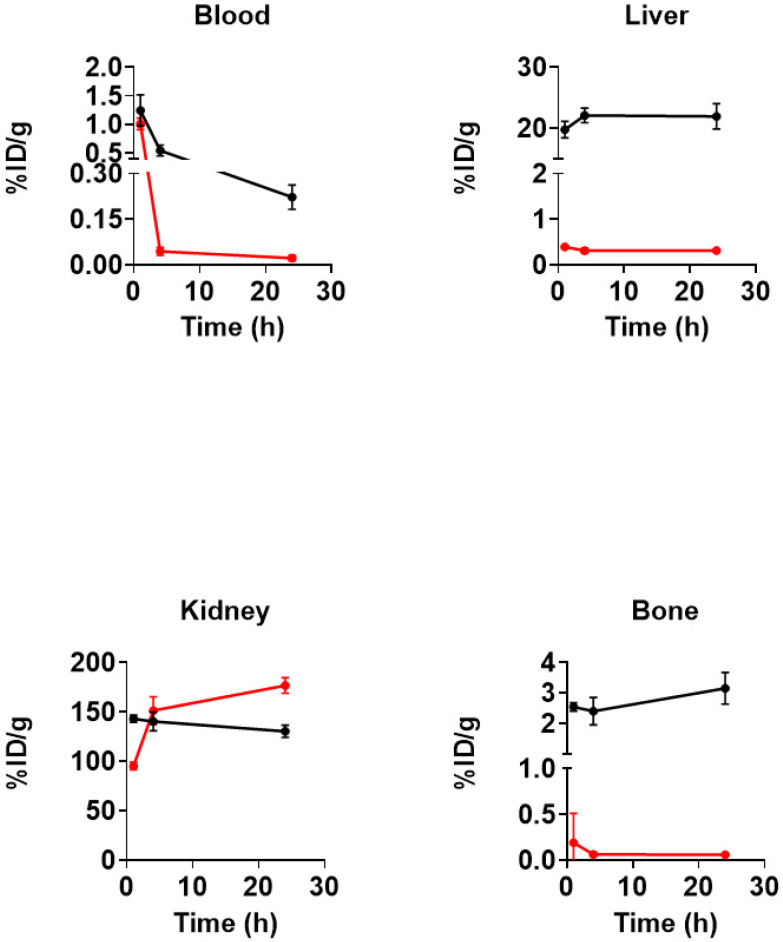
Biodistribution results of [^89^Zr]Zr-A11 (●; red symbol and line) and [^89^Zr]Zr-A12 (●; black symbol and line) in selected tissues. Tumor-bearing mice (*n* = 4 or 5 per group) were injected with either [^89^Zr]Zr-A12 or [^89^Zr]Zr-A11 via the tail vein.

**Figure 3:**
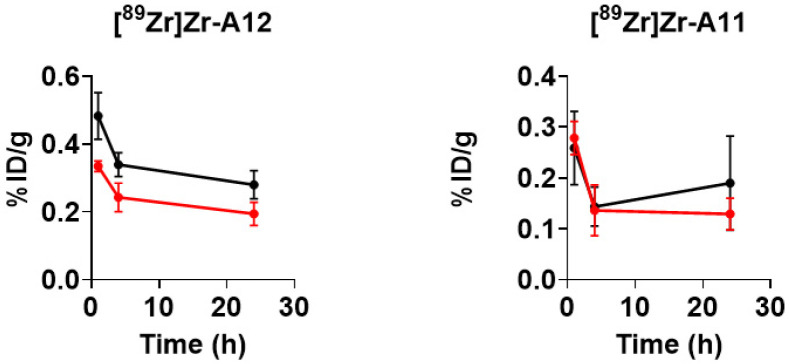
Retention of [^89^Zr]Zr-A11 and [^89^Zr]Zr-A12 in PSMA^+^ LNCAP tumors (●; black symbol and line) and PSMA^−^ PC-3 tumors (●; red symbol and line). Tumor-bearing mice (*n* = 4 or 5 per group) were injected with either [^89^Zr]Zr-A12 or [^89^Zr]Zr-A11 via the tail vein. When comparing the radioactivity retention at 1 h, 4 h and 24 h, animals receiving [^89^Zr]Zr-A12 had significantly more radioactivity associated with PSMA^+^ tumors than PSMA^−^ tumors at 1h (*p* = 0.002), 4h (*p* = 0.004) and 24h (*p* = 0.003). Contrarily, when comparing the radioactivity retention at 1 h, 4 h and 24 h for animals receiving [^89^Zr]Zr-A11, the retention of radioactivity in PSMA^+^ and PSMA^−^ tumors was non-significant at 1 h (*p* = 0.6), 4 h (*p* = 0.8) and 24 h (*p* = 0.3).

In these studies, tumor-bearing animals were randomly divided into cohorts and received a single bolus injection of either [^89^Zr]Zr-A12 or [^89^Zr]Zr-A11. The results are summarized in Figure 2 and Figure 3, while the complete biodistribution data is presented in Tables S1 and S2. The latter radiopharmaceutical was studied to understand the influence that monovalent SA may have on the biodistribution of [^89^Zr]Zr-A12. Animals tolerated both radiopharmaceuticals well with no signs of distress throughout the experimental time course. [^89^Zr]Zr-A11 and [^89^Zr]Zr-A12 demonstrated efficient clearance from the blood pool, with [^89^Zr]Zr-A11 being removed from the systemic circulation more rapidly than [^89^Zr]Zr-A12. For example, [^89^Zr]Zr-A11 demonstrated rapid blood clearance, with 98% of the activity present at 1 h removed from the blood by 24 h. Conversely, [^89^Zr]Zr-A12 demonstrated slower clearance from the systemic circulation, with 82% of the radioactivity present at 1 h removed from the blood by 24 h. This difference in clearance from the systemic circulation most likely reflects the lower molecular weight of the former agent. The radioactivity associated with liver tissue in animals injected with [^89^Zr]Zr-A11 was observed to be low, and it decreased slowly over time; only 20% of the 1 h. activity was excreted by 24 h p.i. This was not the case for animals injected with [^89^Zr]Zr-A12. The radioactivity associated with the liver tissue of animals receiving [^89^Zr]Zr-A12 was elevated at 1 h p.i. and increased by 11% over the experimental time course. Considering the larger molecular weight of [^89^Zr]Zr-A12, it was not surprising to see the retention of radioactivity in the liver when compared to the amount of radioactivity associated with the liver tissue of mice injected with [^89^Zr]Zr-A11. Further, zirconium-89 is a radionuclide that is reported to residualize in tissues [66,85]. Thus, the fact that clearance of radioactivity over the 24 h time course did not occur in either case may reflect the metabolism of both agents in the liver and a residualization of radioactivity within the liver tissue. This explanation would also be consistent with the elevated bone uptake as ^89^Zr is known to localize in bone when not properly chelated or the radiopharmaceutical is metabolized and releases the ^89^Zr from the bioconjugate. However, we did not perform metabolism studies to test this hypothesis. The radioactivity associated with kidney tissue in animals injected with either radiopharmaceutical was elevated. For example, radioactivity associated with the kidney tissue of animals injected with [^89^Zr]Zr-11 in the kidney was extremely high at 1 h p.i. and increased by 85% at 24 h p.i. Likewise, animals injected with [^89^Zr]Zr-12 demonstrated increased radioactivity associated with kidney tissues, but unlike the animals receiving [^89^Zr]Zr-11, radioactivity did decrease slowly over the experimental time course; 91% of the activity observed at 1 h p.i. was still present at 24 h p.i. The excessive amount of radioactivity that was retained in the kidneys most likely reflects the presence of SA. Previously, Schechter et al. evaluated the biodistribution of [^125^I]I-SA in normal, non-tumor bearing balb/c mice. After injection, much of the radiopharmaceutical was observed to localize within the kidney medulla and cortex. Excretion was observed to be slow and occurred over the course of several days [86].

Next, we evaluated the uptake and retention of radioactivity in PSMA^+/−^ tumor tissues in mice receiving either radiopharmaceutical. Although tumor necrosis independent of tumor size led to variable amounts of radioactivity retained in tumors, the biodistribution data corroborated the in vitro data. Mice injected with [^89^Zr]Zr-A12 demonstrated significantly more radioactivity associated with LNCAP tumors when compared to the PC-3 tumors (24 h p.i.; PSMA^+^ vs. PSMA^−^ (%ID/g); *p* value: 0.28 ± 0.042 vs. 0.19 ± 0.034; 0.003); this difference is believed to result from a specific ligand–receptor interaction between [^89^Zr]Zr-A12 and the PSMA protein expressed on the surface of LNCAP cells. Contrarily, there was no statistical difference in the amount of radioactivity associated with the LNCAP or PC-3 tumors when mice were injected with [^89^Zr]Zr-A11 (24 h p.i.; PSMA^+^ vs. PSMA^−^ (%ID/g); *p* value: 0.19 ± 0.092 vs. 0.13 ± 0.031; 0.26).

Finally, when comparing the uptake of [^89^Zr]Zr-A11 and [^89^Zr]Zr-A12 in PSMA^+^ tumors, we observed significantly more tissue-associated radioactivity in the PSMA^+^ tumors of mice injected with [^89^Zr]Zr-A12 at 1 h when compared to the tissue-associated radioactivity in the PSMA^+^ tumors of mice injected with [^89^Zr]Zr-A11 (PSMA^+^ tumors, 1 h p.i.; [^89^Zr]Zr-A12 vs. [^89^Zr]Zr-A11 (%ID/g); *p* value: 0.48 ± 0.068 vs. 0.26 ± 0.072; 0.001). This same phenomenon was also observed at 4 h p.i. (PSMA^+^ tumors, 4 h p.i.; [^89^Zr]Zr-A12 vs. [^89^Zr]Zr-A11 (%ID/g); *p* value: 0.34 ± 0.035 vs. 0.14 ± 0.038; 0.0001). However, by 24 h p.i. the retention of radioactivity in the tumors was not observed to be significantly different (PSMA^+^ tumors, 24 h p.i.; [^89^Zr]Zr-A12 vs. [^89^Zr]Zr-A11 (%ID/g); *p* value: 0.28 ± 0.041 vs. 0.19 ± 0.092; 0.067). This data suggests that the optimum time for PET imaging with [^89^Zr]Zr-A12 may be early time points between 1 h and 4 h p.i. However, one cannot discount the idea that as [^89^Zr]Zr-A12 and [^89^Zr]Zr-A11 circulate within the blood pool they localize in non-target tissues where they begin to be metabolized over time. These non-target tissues then act as reservoirs for radioactive metabolites, which are reintroduced into the circulation and accumulate within tumors because of perfusion effects. However, as metabolism studies were beyond the scope of this work, we did not rigorously test this hypothesis.

### 2.5. Study Limitations and Future Directions

Several limitations to these studies should be considered. Despite best efforts, we were unable to generate an aptamer that had the chelator Df directly attached to one of the DNA strands in sufficient quantity that would facilitate radiochemistry. Further, our attempts to contract with industry to produce the DAC with an appropriate chelator were unsuccessful. Thus, with limited options at the time of these studies, we adopted a more modular approach using the high-affinity interaction between streptavidin and biotin. We chose this method because it facilitated our BLI studies and because the SA–biotin system is inert to a variety of environmental conditions associated with radiochemistry. This approach greatly improved our radiochemical yield, purity, and specific activity.

Despite improving our studies in vitro, however, the incorporation of the SA–biotin complex into our radiopharmaceutical design is believed to have negatively impacted the retention and clearance of radioactivity from biological tissues. Although it was hoped that the chemical properties of the dimeric aptamer complex and the molecular weight of the resulting radiopharmaceutical would dictate the pharmacokinetics of [^89^Zr]Zr-A12, the influence of SA was evident given the excessive retention of radioactivity within kidney tissues, which is consistent with other radiolabeled SA agents [86]. A possible way to mitigate this issue would be to pre-inject non-radioactive SA into the animals before radiopharmaceutical injection. However, we did not attempt this since tumor targeting was modest (vide supra). Additional ways to mitigate the observed excessive kidney uptake would be to adopt bioconjugate synthesis strategies that are reliant on enzyme-mediated chemical modifications of individual nucleic acids, which would then be more amenable to direct conjugation of the ^89^Zr chelator [87]. Click chemistry would also be another reliable way to synthesize the bioconjugate, provided the reactive functional groups can be easily incorporated into the nucleic acid strands [88]. Additionally, radioactivity retention within the liver was also elevated, reflecting hepatobiliary clearance, and this is consistent with other large protein-based radiopharmaceuticals such as the [^89^Zr]Zr-DFO-J591 mAb [89]. Several strategies have been used to reduce excessive radioactivity accumulation in the liver. Previously, poly(ethylene) glycol linkers inserted between the radiometal complex and targeting ligand have reduced hepatic uptake [90,91]. Triglycyl-L-phenylthiourea, a hepatic endopeptidase-sensitive peptide, can also be used as a linker between the radiometal chelate and the dimeric aptamer complex to reduce radioactivity accumulation in liver tissue [92,93]. Finally, while we did see more radioactivity retained in PSMA^+^ tumors when compared to PSMA^−^ tumors, the overall uptake was low when compared to other aptamer systems, peptide based systems such as [^68^Ga]Ga-PSMA-11, or antibody-based agents such as the [^89^Zr]Zr-DFO-J591 mAb and would not warrant clinical translation of [^89^Zr]Zr-A12 without significant chemical modifications that alter its pharmacokinetics in a positive way [49,52,89,94]. One possible reason for this may be that the dimeric aptamer complex used in these studies was identified using in vitro SELEX. Although this method has been used to identify a variety of aptamers for various biomarkers, it requires the aptamers to interact with their target proteins in their native structure. However, the obtained aptamers may not retain those binding characteristics once in vivo since the aptamer’s target on the cell surface may adopt a different conformation than that encountered in the selection process [53]. Further, the biological environment surrounding the target protein may also have affected binding in a negative way. Thus, additional studies that include in silico design or in vivo SELEX using the original DAC as a lead may yield second-generation ligands with improved pharmacokinetics and tumor targeting [52,53,95]. Finally, using ^89^Zr in radiopharmaceutical development may seem an erroneous decision since the excretion pattern of [^89^Zr]Zr-A12 is rapid. However, we chose ^89^Zr as the PET isotope because of our desire to create a PET radiopharmaceutical to estimate dosimetry in advance of alpha particle or beta particle therapy using the same PSMA-specific dimeric aptamer complex as the targeting ligand. While the radioactive half-life of ^89^Zr and the biological half-life of the dimeric aptamer complex may seem mismatched, recent publications have demonstrated the utility of using ^89^Zr in radiopharmaceuticals that rely on rapidly excreting peptides for PCa tumor targeting [77,78,79,80]. Thus, because of these developments and the robust radiochemistry surrounding the use of ^89^Zr, we felt its use in this context was appropriate [67,68,71,72,73].

## 3. Materials and Methods

### 3.1. Reagents and Equipment

Zirconium-89 (^89^Zr: t_½_ = 78.4 h, β^+^: 22.8%) was purchased from Washington University School of Medicine (St. Louis, MO, USA) [83]. Unless noted, all other chemicals and materials were purchased from Sigma-Aldrich Chemical Co. (St. Louis, MO, USA) or ThermoFisher Scientific, Inc. (Waltham, MA, USA). Solutions were prepared using ultrapure water (18 MΩ-cm resistivity). p-isothiocyanatobenzyl-desferrioxamine (Df-Bz-NCS) was purchased from Macrocyclics, Inc. (Dallas, TX, USA). Radiochemistry reaction progress and purity were monitored using an analytical high-performance liquid chromatography (HPLC) system (Waters, Milford, MA, USA), which runs Empower v.3.9.0 software and is configured with a 1525 binary pump, 2707 autosampler, 2998 photodiode array detector, 1500 column heater, fraction collector, size exclusion Superdex 200 10/300 GL (GE Healthcare Life Sciences, Piscataway, NJ, USA) column, a Carrol Ramsey 105 s radioactivity detector (Berkeley, CA, USA), and an isocratic mobile phase (0.5 mL/min) consisting of phosphate buffered saline (PBS, pH 7.1 (NaCl 150 mM, Na_2_HPO_4_ 50 mM, NaH_2_PO_4_ 50 mM, and NaN_3_ 10 mM)). Radio-TLC analysis was performed on a Bioscan AR 2000 radio-TLC scanner equipped with a 10% methane:argon gas supply, a PC interface running Winscan v.3 analysis software (Eckert & Ziegler, Berlin, Germany), Varian ITLC-SG strips (Agilent Technologies, Santa Clara, CA, USA), and 50 mM EDTA (pH 5) as the eluent. Radioactive samples were counted using a Perkin Elmer 2480 Wizard^®^ gamma counter (Revvity, Inc., Waltham, MA, USA) with an energy window of 500–1500 keV. The poly-L-lysine-coated Corning BioCoat coverslips were obtained from ThermoFisher Scientific, Inc. The PSMA-positive cell line LNCaP (CRL-1740) and PSMA-negative cell line PC-3 (CRL-1435) were procured from ATCC (Manassas, VA, USA). The Cy3-labeled DAC (A4), Cy5-labeled DAC (A5), Cy3-labeled dA16 oligo (A7) and the Cy5-labeled dT16 oligo (A8) were purchased from Aptagen, LLC (Jacobus, PA, USA) with a purity of greater than 95%. All other aptamers were synthesized at Integrated DNA Technologies (IDT; Coralville, IA, USA). A table of all relevant aptamers and molecules used in these studies is presented in Table 1.

### 3.2. Synthesis and Purification of Biotinylated Dimeric Aptamer (A10)

Aptamers A2 and A9 were separately reconstituted in nuclease-free water at a final concentration of 100 µM, mixed in a 1:1 ratio, heated to 95 °C for 1 min, and then allowed to anneal at room temperature for 8 h. to form the biotinylated dimeric aptamer (A10). Purification of A10 was accomplished by 12% native polyacrylamide gel electrophoresis (PAGE) or size exclusion (SE) chromatography as previously described [75]. For samples purified by SE-HPLC, aliquots were injected on an SE-HPLC apparatus equipped with a size exclusion Superdex 200 Increase 10/300 GL (code No. 28-9909-04, GE Healthcare Life Sciences, Piscataway, NJ, USA) column and an isocratic mobile phase consisting of phosphate buffered saline (DPBS, catalog No. 17-512F, Lonza, Basel, Switzerland) with a flow rate of 0.5 mLmin^−1^. Fractions (R_t_ = 23.5 to 27.3 min, as shown in Figure S2) were collected and combined. The purified A10 was stored at 4 °C and used for conjugation reactions. The purity of A10 was determined to be greater than 99.8%, and the yield was observed to be 49.8 ± 0.9%.

### 3.3. Synthesis, Purification and Characterization of Desferrioxamine-Conjugated Monovalent Streptavidin (Df-SA; A11)

Df-SA (A11) was prepared using a reported procedure with modifications [67]. Briefly, monovalent SA (10 mg) was dissolved in saline (900 µL), and the pH was adjusted to pH 8.9-9.1 with 0.1 M Na_2_CO_3_ (100 µL). A five-fold molar excess of Df-Bz-NCS (2.43 mg in 15 μL DMSO) was added, and the resulting solution was incubated for 30 min at 37 °C using a thermomixer at 550 r.p.m. To remove non-conjugated Df-Bz-NCS, the reaction solution was purified by a PD-10 column using saline (0.9% NaCl) as the eluent. The A11 complex was stored at 4 °C for subsequent [^89^Zr]Zr-radiochemistry. Characterization of A11 was accomplished using a AXIMA Confidence MALDI-TOF mass spectrometer (Shimadzu Scientific Instruments, Inc., Columbia, MD, USA) and a 6-aza-2-thiothymine (ATT) matrix as previously described [96].

### 3.4. Preparation of Aptamer A12

A two-fold molar excess of Df-SA (A11; 3.47 mg, 694 μL PBS) was added to a solution of biotinylated dimeric aptamer (A10; 5 mg, 500 μL PBS), and the resulting solution was incubated for 2 h. at 30 °C using a thermomixer at 550 r.p.m. To remove unreacted A11, the reaction mixture was purified by SE-HPLC equipped with a size exclusion Superdex 200 Increase 10/300 GL (code No. 28-9909-04, GE Healthcare Life Sciences, Piscataway, NJ, USA) column and an isocratic mobile phase consisting of phosphate buffered saline (DPBS, Lonza, catalog No. 17-512F) with a flow rate of 0.5 mLmin^−1^. Fractions (R_t_ = 21.5 to 29.0 min, as shown in Figure S5) were collected and combined. The purified A12 conjugate was stored at 4 °C and used for [^89^Zr]Zr-radiochemistry.

### 3.5. Mass Photometry (MP) of Aptamers A10 and A12

MP experiments were performed on a Refeyn TwoMP mass photometer (Refeyn Ltd., Oxford, UK). Microscope coverslips (24 mm × 50 mm, Thorlabs Inc., Newton, NJ, USA) were cleaned by serial rinsing with Milli-Q water and HPLC-grade isopropanol Sigma-Aldrich Chemical Co. (St. Louis, MO, USA) followed by drying with a filtered air stream. Cleaned coverslips were then functionally coated with poly-L-lysine (PLL, P4832, Sigma-Aldrich Chemical Co. (St. Louis, MO, USA)). Silicon gaskets (Grace Bio-Labs, Bend, OR, USA) to hold the sample drops were cleaned using the same procedure immediately prior to measurement. All MP measurements were performed at room temperature. The instrument was calibrated using a protein standard mixture of β-amylase (Sigma-Aldrich, 56, 112 and 224 kDa) and thyroglobulin (670 kDa, Sigma-Aldrich Chemical Co. (St. Louis, MO, USA)). Before each measurement, 15 µL of buffer was placed in the well to find focus. The focus position was searched and locked using the default droplet-dilution autofocus function, after which 5 µL of aptamer sample (final concentration of 10 nM) was added and pipetted up and down to briefly mix before movie acquisition was promptly started. Movies were acquired for 1 min using AcquireMP (version 2024-R2; Refeyn, Ltd., Oxford, UK) using standard settings. All movies were processed and analyzed using DiscoverMP (version 2024-R2; Refeyn Ltd., Oxford, UK). Measurements were made in duplicate.

### 3.6. Cell Culture and Confocal Microscopy

LNCaP cells and PC-3 cells were maintained in RPMI 1640 or DMEM/F12 complete media, respectively, and grown at 37 °C in an incubator maintaining an atmosphere containing 5% CO_2_. For microscopy experiments, cells were seeded at 70,000 cells/well in 1.5 mL appropriate media in 12-well plates containing poly-L-lysine-coated Corning BioCoat coverslips, and cells were incubated at 37 °C and 5% CO_2_ for 48 h. Cells were then incubated with either Dulbecco’s PBS buffer control or 1 µM of A6 or A7 for 2 h. Subsequently, the cells were washed with fresh media and D-PBS. This was followed by fixation of the cells with 4% formaldehyde for 5 min at room temperature and multiple washings first with D-PBS followed by Hank’s balanced salt solution (HBSS; Gibco, Waltham, MA, USA). To visualize the cell membrane and the nucleus, cells were stained with wheat germ agglutinin conjugated with Alexa 488 and DAPI, respectively, followed by washing with D-PBS. Microscopy was conducted with a SP8 confocal microscope (Leica Microsystems, Boston, MA, USA).

### 3.7. Binding Affinity Measurement Using Biolayer Interferometry (BLI)

Affinity measurements were performed in 50 mM Tris, 150 mM sodium chloride, and 5 mM magnesium chloride buffer pH 7.5 containing 0.1% BSA using Octet Red 96 (Sartorius, Goettingen, Germany). The purified biotinylated dimeric DNA aptamer (A10) was immobilized on streptavidin-coated biosensors (Sartorius, Goettingen, Germany) for 60 s at the ligand-limiting concentration of 25 nM. Next, the binding rate was obtained by flowing the human PSMA protein as an analyte over the A10-coated biosensor using a dynamic concentration range of 0–50 nM. The rate of dissociation was measured by allowing binding buffer to pass over the antigen–A10 complexes immobilized on the biosensor. Curves were fit using a 1:1 binding model equation after background correction. The binding assay was performed in triplicate, and affinities were calculated from association and dissociation rates using ForteBio Data analysis software v11.1. The values are represented as means ± standard errors.

### 3.8. Animal Care and Use

All studies were carried out in strict accordance with the recommendations in the Guide for the Care and Use of Animals of the National Institutes of Health and approved by the University of Iowa’s Institutional Animal Care and Use Committee (Protocol # 302265-001). Five-week-old athymic nude mice (Inotiv, West Lafayette, IN, USA) were maintained according to the Animal Welfare Act, the guidelines established by the NIH and all other federal and state statutes and regulations pertaining to laboratory animal care. All animals were acclimated to a 12 h light/dark cycle and received food and water ad libitum. All animals were checked by veterinary staff daily to ensure well-being and proper environment and were monitored for signs of distress. Clinical signs of distress in laboratory rodents include decreased activity, pilo-erection, un-groomed appearance, excessive licking and scratching, self-mutilation, abnormal stance, hunched appearance, rapid or shallow respiration, grunting, dilated pupils, aggressiveness towards handlers, high-pitch vocalizations, change in feeding activity, and attempts to separate from the group. In the event of clinical distress, animals were euthanized after consulting the on-call veterinarian. To minimize suffering during the tail vain injection, animals were kept under deep isoflurane anesthesia. At the end point of the study, animals were anesthetized with isoflurane and were euthanized by cervical dislocation, which is consistent with the recommendations of the American Veterinary Medical Association Guidelines on Euthanasia. Animals were monitored every 30 min during the experiment, but during the experiment, none of the animals died or were found ill before the experimental end point.

### 3.9. Tumor Xenograft Implantation

Tumors were formed by the subcutaneous injection of either LNCaP or PC-3 tumor cells (5 × 10^6^ cells; mixed 1:1 in Matrigel^®^ matrix basement membrane (Corning, Inc., Glendale, AZ, USA)) in contralateral flanks. The total volume of each injection was 100 µL. Cell growth was evaluated weekly using manual tumor volume (volume = 0.52 × [width]^2^ × [length]) measurements. Only animals bearing tumors greater than 100 mm^3^ were used in further studies.

### 3.10. Preparation of [^89^Zr]Zr-A11

[^89^Zr]Zr-oxalate (1.4–1.6 mCi, 50–60 MBq in 20–25 µL, 0.1 M oxalic acid) was placed in a 1.5 mL tube and the pH was adjusted to 6.8–7.2 using 0.5 M HEPES buffer (700 μL, pH 7.2). Next, sodium acetate buffer (NaOAc; 200 µL, 0.25 M, pH 6.8–7.0) containing n-acetyl-L-cysteine (NAC; 5 mgmL^−1^; pH 6.8–7.0) and 350 µg of A11 (70 µL saline) were added, followed by a 30 min incubation period at 22 °C. The crude reaction mixture was purified using a PD-10 column (eluent: NAC, 5 mgmL^−1^ in 0.25 M sodium acetate buffer, pH 6.8–7.0). Radiochemical yield and purity of [^89^Zr]Zr-A11 were determined by radio-TLC and radio-HPLC as previously described [67].

### 3.11. Preparation of [^89^Zr]Zr-A12

[^89^Zr]Zr-oxalate (1.6–1.8 mCi, 59–67 MBq in 50–55 µL, 0.1 M oxalic acid) was placed in a 1.5 mL tube and the pH was adjusted to 6.8–7.2 using 0.5 M HEPES buffer (700 μL, pH 7.2). Next, sodium acetate buffer (NaOAc; 200 µL, 0.25 M, pH 6.8–7.0) containing n-acetyl-L-cysteine (NAC; 5 mgmL^−1^; pH 6.8–7.0), DMSO (20 µL) and 380 µg of A12 (100 µL PBS) were added, and the resulting solution was incubated at 75 °C for 15 min followed by a 30 min incubation period at room temperature. The crude reaction mixture was purified using a PD-10 column (eluent: NAC, 5 mgmL^−1^ in 0.25 M sodium acetate buffer, pH 6.8–7.0). Radiochemical yield and purity of [^89^Zr]Zr-A12 were determined by radio-TLC and HPLC as previously described [72].

### 3.12. In Vitro Serum Stability of [^89^Zr]Zr-A11

In vitro serum stability was carried out by adding 90 μCi (3.3 MBq) of [^89^Zr]Zr-A11 (60 µL, NaOAc containing NAC, 0.5 mgmL^−1^, pH 6.8–7.0) to 500 µL human serum. The solutions (n = 3) were incubated at 37 °C for 7 days and analyzed at 1, 3, 5 and 7 days by radio-TLC using a mobile phase consisting of 50 mM EDTA (pH 5) on Agilent ITLC-SG strips (Agilent Technologies, Santa Clara, CA, USA). The percent (%) of intact [^89^Zr]Zr-A11 was calculated as described previously [72].

### 3.13. In Vitro Serum Stability of [^89^Zr]Zr-A12

In vitro serum stability was carried out by adding 95 μCi (3.52 MBq) of [^89^Zr]Zr-A12 (50 µL, NaOAc containing NAC, 0.5 mgmL^−1^, pH 6.8–7.0) to 500 µL human serum. The solutions (n = 3) were incubated at 37 °C for 7 days and analyzed at 1, 3, 5 and 7 days by radio-TLC using a mobile phase consisting of 50 mM EDTA (pH 5) on Agilent ITLC-SG strips. The percent (%) of intact [^89^Zr]Zr-A12 was calculated as described previously [72].

### 3.14. Acute Biodistribution Studies in Tumor-Bearing Mice

Tumor-bearing mice (n = 4 or 5 per group) were injected with either [^89^Zr]Zr-A12 (15 μCi, 555 kBq; 150 µL PBS/mouse) or [^89^Zr]Zr-A11 (15 μCi, 555 kBq; 150 µL PBS/mouse) via the tail vein. Mice were euthanized at 1, 4, and 24 h post-injection (p.i.). Blood, tumors and tissues of interest were removed, weighed and subjected to gamma counting. The percent injected dose per gram (%ID/gram) was calculated by comparison to a weighed, counted standard.

### 3.15. Statistical Methods

All data are presented as means ± SDs or means (95% confidence intervals). For statistical classification, a Student’s *t* test (two-tailed, unpaired) was performed using GraphPad Prism software version 9.5.1. (GraphPad Software, Boston, MA, USA). Any difference of *p* < 0.05 was considered significant.

## 4. Conclusions

In summary, this work evaluated a dimeric aptamer complex which is selective for PSMA^+^ prostate cancer cells and is amenable to derivatization with PET radioisotopes. Confocal microscopy confirmed the selective nature of the dimeric aptamer complex for PSMA^+^ LNCAP tumor cells, while BLI revealed that the dimeric aptamer complex had a high affinity for the PSMA protein that was consistent with other PSMA-targeting biomolecules. In addition, the radiolabeled analog [^89^Zr]Zr-A12 was prepared and evaluated in acute biodistributions studies. Elevated levels of radioactivity in PSMA^+^ tumors were observed when compared to PSMA^−^ tumors. However, radioactivity retention in the kidney was excessive due to the presence of streptavidin. While promising, future improvements in radiopharmaceutical design will be required to enhance tumor uptake while reducing kidney retention before clinical translation can be attempted.

## 5. Patents

WHG has been awarded patent US 9,486,533 B2 relating to this work.

## Figures and Tables

**Table 1:** Aptamer Variants Used in these Studies.

Aptamer Number	Aptamer Name	Sequence/Notes	Ref.
A1	Anti-PSMA strand 1	5′-GCG TTT TCG CTT TTG CGT TTT GGG TCA TCT GCT TAC GAT AGC AAT GCT CGG CAA AAA AAA AAA AAA AAG CCG-3′	[59]
A2	Anti-PSMA strand 2	5′-GCG TTT TCG CTT TTG CGT TTT GGG TCA TCT GCT TAC GAT AGC AAT GCT CGG CTT TTT TTT TTT TTT TTG CCG-3′	[59]
A3	DAC	Annealed A1 and A2	[59]
A4	Cy3-A1	5′-Cy3-GCG TTT TCG CTT TTG CGT TTT GGG TCA TCT GCT TAC GAT AGC AAT GCT CGG CAA AAA AAA AAA AAA AAG CC-invG-3′	This work
A5	Cy5-A2	5′-Cy5-GCG TTT TCG CTT TTG CGT TTT GGG TCA TCT GCT TAC GAT AGC AAT GCT CGG CTT TTT TTT TTT TTT TTG CC-invG-3′	This work
A6	Cy3-Cy5 labeled DAC	Annealed aptamer A4 and A5	This work
A7	Cy3-dA16	5′-Cy3-AAA AAA AAA AAA AAA A-3′	This work
A8	Cy5-dT16	5′-Cy5-TTT TTT TTT TTT TTT T-3′	This work
A9	Biotinylated anti-PSMA strand 1	5′-Biotin-A1-3′	This work
A10	BioDAC	Annealed A9 and A2	This work
A11	Df-SA	Desferrioxamine-modified monovalent streptavidin	This work
A12	Df-SA-BioDAC	A11 conjugated to A10	This work

**Table 2:** Chronological In Vitro Serum Stability Study of [^89^Zr]Zr-A11 and [^89^Zr]Zr-A12 in Human Serum Maintained at 37 °C for 7 days as Determined by Radio-TLC ^1^.

Time Point (h)	Percent (%) Intact [^89^Zr]Zr-A11	Percent (%) Intact [^89^Zr]Zr-A12
0	99.8 ± 0.2	99.8 ± 0.2
24	99.2 ± 0.4	98.7 ± 0.5
72	98.5 ± 0.3	98.1 ± 0.2
120	97.8 ± 0.1	97.3 ± 0.3
168	97.2 ± 0.3	95.6 ± 0.4

^1^ Data is the average of three replicates.

## Data Availability

The datasets used and/or analyzed during the current study are available from the corresponding author upon reasonable request.

## Supplementary Materials

The following supporting information can be downloaded at: https://www.mdpi.com/article/10.3390/molecules31030493/s1, radiochemistry data, in vitro data, and in vivo data. A 12% native SDS-PAGE gel depicting aptamers A2, A9 and A10 (Figure S1). SE-HPLC purification of A10 (Figure S2). MALDI-MS spectrum of A11 (Figure S3). Mass photometry spectrum of A10 (Figure S4). Mass photometry spectrum of A12 (Figure S5). Confocal microscopy imaging demonstrates the dimeric aptamer complex binds to PSMA+ LNCAP cells (Figure S6). Biolayer interferometry analysis of the dimeric aptamer complex, A10 for PSMA protein (Figure S7). Size exclusion HPLC purification of A12 (Figure S8). Quality control of [^89^Zr]Zr-A12 using radio-TLC (Figure S9). Quality control of [^89^Zr]Zr-A12 using radio-HPLC (Figure S10). Stability of [^89^Zr]Zr-A12 in human serum as assessed by Radio-ITLC (Figure S11). Quality control of [^89^Zr]Zr-A11 using radio-TLC (Figure S12). Quality control of [^89^Zr]Zr-A11 using radio-HPLC (Figure S13). Stability of [^89^Zr]Zr-A11 in human serum at 37֯C as assessed by Radio-TLC (Figure S14). Complete biodistribution of [^89^Zr]Zr-A12 (Table S1). Complete biodistribution of [^89^Zr]Zr-A11 (Table S2).

## Abbreviations

ATT: 6-aza-2-thiothymine; Df: Desferrioxamine; PCTA: 3,6,9,15-Tetraazabicyclo [9.3.1]pentadeca-1(15),11,13-triene-3,6,9-triacetic acid; NOTA: 2,2′,2″-(1,4,7-triazacyclononane-1,4,7-triyl)triacetic acid; TRITA: 2,2′,2″,2‴-(1,4,7,10-tetraazacyclo tridecane-1,4,7,10-tetrayl)tetraacetic acid; TETA: 2,2′,2″,2‴-(1,4,8,11-tetraazacyclotetradecane-1,4,8,11-tetrayl)tetraacetic acid; EDTA: Ethylenediaminetetraacetic acid; Zr(AcAc)_4_: Zirconium(IV) acetylacetonate; Zr(ox)_2_: Zirconium(IV) Oxalate; TFA: Trifluoroacetic acid; MeOH: Methanol; NH_4_Cl: Ammonium chloride; NH_4_OAc: Ammonium acetate; NaOAc: Sodium acetate; TMAA: Tetramethylammonium acetate; MES: 2-(N-morpholino)ethanesulfonic acid; HEPES: 4-(2-Hydroxyethyl) piperazine-1-ethanesulfonic acid; TRIS: Tris(hydroxymethyl)aminomethane; NMR: Nuclear Magnetic Resonance; HMBC: Heteroatom Multiple-Bond Correlation; HMQC: Heteronuclear Multiple Quantum Coherence; UV-HPLC: Ultra-Violet High Performance Liquid Chromatography; RT: Retention Time; R_f_: Retention Factor; ESI-HR-MS: Electrospray Ionization High Resolution Mass Spectrometry; HS: Human Serum; ITLC: Instant Thin Layer Chromatography; kBq: Kilobecquerel; MBq: Megabecquerel; %ID/g: Percent Injected Dose Per Gram; P.I.: Post-injection; ROI: Region of Interest; CPM: Counts per Minute; TLC: Thin Layer Chromatography; PET: Positron Emission Tomography; CT: Computed Tomography; PSMA: Prostate-Specific Membrane Antigen; WGA: Wheat Germ Agglutinin; DAC: Dimeric Aptamer Complex; BioDAC: Biotinylated Dimeric Aptamer Complex; BLI: Bio-Layer Interferometry; SA: Streptavidin; ^89^Zr: Zirconium-89.

## References

[1] Rebello R.J., Oing C., Knudsen K.E., Loeb S., Johnson D.C., Reiter R.E., Gillessen S., Van der Kwast T., Bristow R.G. (2021). Prostate cancer. Nat. Rev. Dis. Primers.

[2] Siegel R.L., Miller K.D., Jemal A. (2018). Cancer statistics, 2018. CA Cancer J. Clin..

[3] Tu S.M., Lin S.H. (2008). Current trials using bone-targeting agents in prostate cancer. Cancer J..

[4] Gravestock P., Somani B.K., Tokas T., Rai B.P. (2023). A Review of Modern Imaging Landscape for Prostate Cancer: A Comprehensive Clinical Guide. J. Clin. Med..

[5] Pawson A., Ghumman Z., Kuo P.H., Jadvar H., Bartel T., Shayegan B., Zukotynski K. (2020). A review of prostate cancer imaging, positron emission tomography, and radiopharmaceutical-based therapy. Can. Urol. Assoc. J..

[6] Jadvar H. (2013). Molecular imaging of prostate cancer with PET. J. Nucl. Med..

[7] Jadvar H. (2015). Prognostic Utility of PET in Prostate Cancer. PET Clin..

[8] Jadvar H. (2016). Positron emission tomography in imaging evaluation of staging, restaging, treatment response, and prognosis in prostate cancer. Abdom. Radiol..

[9] Castellucci P., Jadvar H. (2012). PET/CT in prostate cancer: Non-choline radiopharmaceuticals. Q. J. Nucl. Med. Mol. Imaging.

[10] Jadvar H. (2009). Molecular imaging of prostate cancer with 18F-fluorodeoxyglucose PET. Nat. Rev. Urol..

[11] Jadvar H. (2011). Prostate cancer: PET with 1^8^F-FDG, ^18^F-or ^11^C-acetate, and ^18^F- or 11C-choline. J. Nucl. Med..

[12] Jadvar H. (2013). Imaging evaluation of prostate cancer with 18F-fluorodeoxyglucose PET/CT: Utility and limitations. Eur. J. Nucl. Med. Mol. Imaging.

[13] Bogdanovic B., Solari E.L., Villagran Asiares A., van Marwick S., Schachoff S., Eiber M., Weber W.A., Nekolla S.G. (2023). Is there more than meets the eye in PSMA imaging in prostate cancer with PET/MRI? Looking closer at uptake time, correlation with PSA and Gleason score. Eur. J. Hybrid. Imaging.

[14] Liu I.J., Zafar M.B., Lai Y.H., Segall G.M., Terris M.K. (2001). Fluorodeoxyglucose positron emission tomography studies in diagnosis and staging of clinically organ-confined prostate cancer. Urology.

[15] Krause B.J., Souvatzoglou M., Tuncel M., Herrmann K., Buck A.K., Praus C., Schuster T., Geinitz H., Treiber U., Schwaiger M. (2008). The detection rate of [^11^C]choline-PET/CT depends on the serum PSA-value in patients with biochemical recurrence of prostate cancer. Eur. J. Nucl. Med. Mol. Imaging.

[16] Armstrong J.M., Martin C.R., Dechet C., Morton K., Evans D., Ambrose J., Maughan B.L., O’Neil B., Lowrance W. (2020). (18)F-fluciclovine PET CT detection of biochemical recurrent prostate cancer at specific PSA thresholds after definitive treatment. Urologic Oncology: Seminars and Original Investigations.

[17] Suzuki H., Inoue Y., Fujimoto H., Yonese J., Tanabe K., Fukasawa S., Inoue T., Saito S., Ueno M., Otaka A. (2016). Diagnostic performance and safety of NMK36 (*trans*-1-amino-3-[^18^F]fluorocyclobutanecarboxylic acid)-PET/CT in primary prostate cancer: Multicenter Phase IIb clinical trial. Jpn. J. Clin. Oncol..

[18] Haseman M.K., Rosenthal S.A., Polascik T.J. (2000). Capromab Pendetide imaging of prostate cancer. Cancer Biother. Radiopharm..

[19] Chen Y., Pullambhatla M., Foss C.A., Byun Y., Nimmagadda S., Senthamizhchelvan S., Sgouros G., Mease R.C., Pomper M.G. (2011). 2-(3-1-Carboxy-5-[(6-[^18^F]fluoro-pyridine-3-carbonyl)-amino]-pentyl-ureido)-pentanedioic acid, [^18^F]DCFPyL, a PSMA-based PET imaging agent for prostate cancer. Clin. Cancer Res..

[20] Eder M., Schafer M., Bauder-Wust U., Hull W.E., Wangler C., Mier W., Haberkorn U., Eisenhut M. (2012). 68Ga-complex lipophilicity and the targeting property of a urea-based PSMA inhibitor for PET imaging. Bioconjugate Chem..

[21] Pandit-Taskar N., O’Donoghue J.A., Beylergil V., Lyashchenko S., Ruan S., Solomon S.B., Durack J.C., Carrasquillo J.A., Lefkowitz R.A., Gonen M. (2014). ^89^Zr-huJ591 immuno-PET imaging in patients with advanced metastatic prostate cancer. Eur. J. Nucl. Med. Mol. Imaging.

[22] Pandit-Taskar N., O’Donoghue J.A., Durack J.C., Lyashchenko S.K., Cheal S.M., Beylergil V., Lefkowitz R.A., Carrasquillo J.A., Martinez D.F., Fung A.M. (2015). A Phase I/II Study for Analytic Validation of 89Zr-J591 ImmunoPET as a Molecular Imaging Agent for Metastatic Prostate Cancer. Clin. Cancer Res..

[23] Ananias H.J., de Jong I.J., Dierckx R.A., van de Wiele C., Helfrich W., Elsinga P.H. (2008). Nuclear imaging of prostate cancer with gastrin-releasing-peptide-receptor targeted radiopharmaceuticals. Curr. Pharm. Des..

[24] Bakker I.L., Froberg A.C., Busstra M.B., Verzijlbergen J.F., Konijnenberg M., van Leenders G., Schoots I.G., de Blois E., van Weerden W.M., Dalm S.U. (2021). GRPr Antagonist ^68^Ga-SB3 PET/CT Imaging of Primary Prostate Cancer in Therapy-Naive Patients. J. Nucl. Med..

[25] Ceci F., Castellucci P., Polverari G., Iagaru A. (2020). Clinical application of Fluciclovine PET, choline PET and gastrin-releasing polypeptide receptor (bombesin) targeting PET in prostate cancer. Curr. Opin. Urol..

[26] Evangelista L., Briganti A., Fanti S., Joniau S., Reske S., Schiavina R., Stief C., Thalmann G.N., Picchio M. (2016). New Clinical Indications for ^18^F/(11)C-choline, New Tracers for Positron Emission Tomography and a Promising Hybrid Device for Prostate Cancer Staging: A Systematic Review of the Literature. Eur. Urol..

[27] Maina T., Nock B.A. (2017). From Bench to Bed: New Gastrin-Releasing Peptide Receptor-Directed Radioligands and Their Use in Prostate Cancer. PET Clin..

[28] Schuster D.M., Nanni C., Fanti S. (2016). PET Tracers Beyond FDG in Prostate Cancer. Semin. Nucl. Med..

[29] Zhang J., Niu G., Fan X., Lang L., Hou G., Chen L., Wu H., Zhu Z., Li F., Chen X. (2018). PET Using a GRPR Antagonist ^68^Ga-RM26 in Healthy Volunteers and Prostate Cancer Patients. J. Nucl. Med..

[30] Santhanam P., Russell J., Rooper L.M., Ladenson P.W., Pomper M.G., Rowe S.P. (2020). The prostate-specific membrane antigen (PSMA)-targeted radiotracer ^18^F-DCFPyL detects tumor neovasculature in metastatic, advanced, radioiodine-refractory, differentiated thyroid cancer. Med. Oncol..

[31] Hofer C., Laubenbacher C., Block T., Breul J., Hartung R., Schwaiger M. (1999). Fluorine-18-fluorodeoxyglucose positron emission tomography is useless for the detection of local recurrence after radical prostatectomy. Eur. Urol..

[32] Afshar-Oromieh A., Babich J.W., Kratochwil C., Giesel F.L., Eisenhut M., Kopka K., Haberkorn U. (2016). The Rise of PSMA Ligands for Diagnosis and Therapy of Prostate Cancer. J. Nucl. Med..

[33] Widjaja L., Werner R.A., Ross T.L., Bengel F.M., Derlin T. (2021). PSMA Expression Predicts Early Biochemical Response in Patients with Metastatic Castration-Resistant Prostate Cancer under ^177^Lu-PSMA-617 Radioligand Therapy. Cancers.

[34] (2021). FDA approves a second PSMA targeting agent for PET imaging in men with prostate cancer. BJU Int..

[35] Aksu A., Karahan Sen N.P., Tuna E.B., Aslan G., Capa Kaya G. (2021). Evaluation of ^68^Ga-PSMA PET/CT with volumetric parameters for staging of prostate cancer patients. Nucl. Med. Commun..

[36] Alberts I., Prenosil G., Mingels C., Bohn K.P., Viscione M., Sari H., Rominger A., Afshar-Oromieh A. (2021). Feasibility of late acquisition [^68^Ga]Ga-PSMA-11 PET/CT using a long axial field-of-view PET/CT scanner for the diagnosis of recurrent prostate cancer-first clinical experiences. Eur. J. Nucl. Med. Mol. Imaging.

[37] Albisinni S., Sarkis J., Diamand R., De Nunzio C. (2023). Prebiopsy ^68^Ga-PSMA PET imaging: Can we improve the current diagnostic pathway for prostate cancer?. Prostate Cancer Prostatic Dis..

[38] Awenat S., Piccardo A., Carvoeiras P., Signore G., Giovanella L., Prior J.O., Treglia G. (2021). Diagnostic Role of ^18^F-PSMA-1007 PET/CT in Prostate Cancer Staging: A Systematic Review. Diagnostics.

[39] Bailey J., Piert M. (2017). Performance of ^68^Ga-PSMA PET/CT for Prostate Cancer Management at Initial Staging and Time of Biochemical Recurrence. Curr. Urol. Rep..

[40] Boegemann M., Schrader A.J., Rahbar K. (2017). ^177^Lu-PSMA therapy: Current evidence for use in the treatment of patients with metastatic prostate cancer. Die Urologie.

[41] Bois F., Noirot C., Dietemann S., Mainta I.C., Zilli T., Garibotto V., Walter M.A. (2020). [^68Ga^]Ga-PSMA-11 in prostate cancer: A comprehensive review. Am. J. Nucl. Med. Mol. Imaging.

[42] Dhiantravan N., Emmett L., Joshua A.M., Pattison D.A., Francis R.J., Williams S., Sandhu S., Davis I.D., Vela I., Neha N. (2021). UpFrontPSMA: A randomized phase 2 study of sequential ^177^ Lu-PSMA-617 and docetaxel vs docetaxel in metastatic hormone-naive prostate cancer (clinical trial protocol). BJU Int..

[43] Ghodsirad M.A., Pirayesh E., Akbarian R., Javanmard B., Kaghazchi F., Tavakoli M., Fattahi K. (2020). Diagnostic Utility of Lutetium-177 (Lu 177) Prostate-Specific Membrane Antigen (PSMA) Scintigraphy in Prostate Cancer Patients with PSA Rise and Negative Conventional Imaging. Urol. J..

[44] Hennrich U., Eder M. (2021). [^68^Ga]Ga-PSMA-11: The First FDA-Approved ^68^Ga-Radiopharmaceutical for PET Imaging of Prostate Cancer. Pharmaceuticals.

[45] Hennrich U., Eder M. (2022). [^177^Lu]Lu-PSMA-617 (Pluvicto(TM)): The First FDA-Approved Radiotherapeutical for Treatment of Prostate Cancer. Pharmaceuticals.

[46] Okamoto S., Thieme A., Allmann J., D’Alessandria C., Maurer T., Retz M., Tauber R., Heck M.M., Wester H.J., Tamaki N. (2017). Radiation Dosimetry for ^177^Lu-PSMA I&T in Metastatic Castration-Resistant Prostate Cancer: Absorbed Dose in Normal Organs and Tumor Lesions. J. Nucl. Med..

[47] Schuchardt C., Zhang J., Kulkarni H.R., Chen X., Muller D., Baum R.P. (2022). Prostate-Specific Membrane Antigen Radioligand Therapy Using ^177^Lu-PSMA I&T and ^177^Lu-PSMA-617 in Patients with Metastatic Castration-Resistant Prostate Cancer: Comparison of Safety, Biodistribution, and Dosimetry. J. Nucl. Med..

[48] Yadav D., Hwang H., Qiao W., Upadhyay R., Chapin B.F., Tang C., Aparicio A., Lopez-Olivo M.A., Kang S.K., Macapinlac H.A. (2022). ^18^F-Fluciclovine versus PSMA PET Imaging in Primary Tumor Detection during Initial Staging of High-Risk Prostate Cancer: A Systematic Review and Meta-Analysis. Radiol. Imaging Cancer.

[49] Cruz-Hernandez C.D., Rodriguez-Martinez G., Cortes-Ramirez S.A., Morales-Pacheco M., Cruz-Burgos M., Losada-Garcia A., Reyes-Grajeda J.P., Gonzalez-Ramirez I., Gonzalez-Covarrubias V., Camacho-Arroyo I. (2022). Aptamers as Theragnostic Tools in Prostate Cancer. Biomolecules.

[50] Lakhin A.V., Tarantul V.Z., Gening L.V. (2013). Aptamers: Problems, solutions and prospects. Acta Naturae.

[51] Zhou J., Rossi J. (2017). Aptamers as targeted therapeutics: Current potential and challenges. Nat. Rev. Drug Discov..

[52] Campos-Fernandez E., Barcelos L.S., Souza A.G., Goulart L.R., Alonso-Goulart V. (2020). Post-SELEX Optimization and Characterization of a Prostate Cancer Cell-Specific Aptamer for Diagnosis. ACS Omega.

[53] Sola M., Menon A.P., Moreno B., Meraviglia-Crivelli D., Soldevilla M.M., Carton-Garcia F., Pastor F. (2020). Aptamers Against Live Targets: Is In Vivo SELEX Finally Coming to the Edge?. Mol. Ther. Nucleic Acids.

[54] Vahidi H., Nafissi-Varcheh N., Kazemi B., Aboofazeli R., Shahhosseini S., Tabarzad M. (2017). Challenges to Design and Develop of DNA Aptamers for Protein Targets. II. Development of the Aptameric Affinity Ligands Specific to Human Plasma Coagulation Factor VIII Using SEC-SELEX. Iran. J. Pharm. Res..

[55] Shigdar S., Macdonald J., O’Connor M., Wang T., Xiang D., Al Shamaileh H., Qiao L., Wei M., Zhou S.F., Zhu Y. (2013). Aptamers as theranostic agents: Modifications, serum stability and functionalisation. Sensors.

[56] Lupold S.E. (2018). Aptamers and apple pies: A mini-review of PSMA aptamers and lessons from Donald S. Coffey. Am. J. Clin. Exp. Urol..

[57] Savory N., Abe K., Sode K., Ikebukuro K. (2010). Selection of DNA aptamer against prostate specific antigen using a genetic algorithm and application to sensing. Biosens. Bioelectron..

[58] Shigdar S., Lin J., Yu Y., Pastuovic M., Wei M., Duan W. (2011). RNA aptamer against a cancer stem cell marker epithelial cell adhesion molecule. Cancer Sci..

[59] Boyacioglu O., Stuart C.H., Kulik G., Gmeiner W.H. (2013). Dimeric DNA Aptamer Complexes for High-capacity-targeted Drug Delivery Using pH-sensitive Covalent Linkages. Mol. Ther. Nucleic Acids.

[60] Jadvar H. (2011). Prostate cancer. Methods Mol. Biol..

[61] Kratochvil J., van Wee R., Thiele J.C., Loewenthal D., Bardzil J., Iqbal K., Benesch J.L.P., Thorpe S., Kukura P. (2025). Best practice mass photometry: A guide to optimal single-molecule mass measurement. Nat. Protoc..

[62] Bates T.A., Gurmessa S.K., Weinstein J.B., Trank-Greene M., Wrynla X.H., Anastas A., Anley T.W., Hinchliff A., Shinde U., Burke J.E. (2025). Biolayer interferometry for measuring the kinetics of protein-protein interactions and nanobody binding. Nat. Protoc..

[63] Concepcion J., Witte K., Wartchow C., Choo S., Yao D., Persson H., Wei J., Li P., Heidecker B., Ma W. (2009). Label-free detection of biomolecular interactions using BioLayer interferometry for kinetic characterization. Comb. Chem. High. Throughput Screen.

[64] Murali S., Rustandi R.R., Zheng X., Payne A., Shang L. (2022). Applications of Surface Plasmon Resonance and Biolayer Interferometry for Virus-Ligand Binding. Viruses.

[65] Smith-Jones P.M., Vallabahajosula S., Goldsmith S.J., Navarro V., Hunter C.J., Bastidas D., Bander N.H. (2000). In vitro characterization of radiolabeled monoclonal antibodies specific for the extracellular domain of prostate-specific membrane antigen. Cancer Res..

[66] Wadas T.J., Wong E.H., Weisman G.R., Anderson C.J. (2010). Coordinating radiometals of copper, gallium, indium, yttrium, and zirconium for PET and SPECT imaging of disease. Chem. Rev..

[67] Bhatt N.B., Pandya D.N., Rideout-Danner S., Gage H.D., Marini F.C., Wadas T.J. (2018). A comprehensively revised strategy that improves the specific activity and long-term stability of clinically relevant ^89^Zr-immuno-PET agents. Dalton Trans..

[68] Bhatt N.B., Pandya D.N., Wadas T.J. (2018). Recent Advances in Zirconium-89 Chelator Development. Molecules.

[69] Bhatt N.B., Pandya D.N., Xu J., Tatum D., Magda D., Wadas T.J. (2017). Evaluation of macrocyclic hydroxyisophthalamide ligands as chelators for zirconium-89. PLoS ONE.

[70] NTinianow J., Pandya D.N., Pailloux S.L., Ogasawara A., Vanderbilt A.N., Gill H.S., Williams S.P., Wadas T.J., Magda D., Marik J. (2016). Evaluation of a 3-hydroxypyridin-2-one (2,3-HOPO) Based Macrocyclic Chelator for ^89^Zr(4+) and Its Use for ImmunoPET Imaging of HER2 Positive Model of Ovarian Carcinoma in Mice. Theranostics.

[71] Pandya D.N., Bhatt N., Yuan H., Day C.S., Ehrmann B.M., Wright M., Bierbach U., Wadas T.J. (2017). Zirconium tetraazamacrocycle complexes display extraordinary stability and provide a new strategy for zirconium-89-based radiopharmaceutical development. Chem. Sci..

[72] Pandya D.N., Bhatt N.B., Almaguel F., Rideout-Danner S., Gage H.D., Solingapuram Sai K.K., Wadas T.J. (2019). ^89^Zr-Chloride Can Be Used for Immuno-PET Radiochemistry Without Loss of Antigen Reactivity In Vivo. J. Nucl. Med..

[73] Pandya D.N., Henry K.E., Day C.S., Graves S.A., Nagle V.L., Dilling T.R., Sinha A., Ehrmann B.M., Bhatt N.B., Menda Y. (2020). Polyazamacrocycle Ligands Facilitate ^89^Zr Radiochemistry and Yield ^89^Zr Complexes with Remarkable Stability. Inorg. Chem..

[74] Pandya D.N., Pailloux S., Tatum D., Magda D., Wadas T.J. (2015). Di-macrocyclic terephthalamide ligands as chelators for the PET radionuclide zirconium-89. Chem. Commun..

[75] Pandya D.N., Sinha A., Yuan H., Mutkus L., Stumpf K., Marini F.C., Wadas T.J. (2020). Imaging of Fibroblast Activation Protein Alpha Expression in a Preclinical Mouse Model of Glioma Using Positron Emission Tomography. Molecules.

[76] Imura R., Ozeki A.N., Shida N., Kobayashi M., Ida H., Wada Y., Akimitsu N., Kumakura Y. (2022). Radiolabeling of PSMA-617 with ^89^Zr: A novel use of DMSO to improve radiochemical yield and preliminary small-animal PET results. Nucl. Med. Biol..

[77] Prive B.M., Derks Y.H.W., Rosar F., Franssen G.M., Peters S.M.B., Khreish F., Bartholoma M., Maus S., Gotthardt M., Laverman P. (2022). ^89^Zr-labeled PSMA ligands for pharmacokinetic PET imaging and dosimetry of PSMA-617 and PSMA-I&T: A preclinical evaluation and first in man. Eur. J. Nucl. Med. Mol. Imaging.

[78] Rosar F., Burgard C., Linxweiler J., Wagner M., Ezziddin S. (2023). Histologically Confirmed Testicular Metastasis Revealed by [^89^Zr]Zr-PSMA-617 PET/CT in a Patient with Biochemical Recurrence of Prostate Cancer and Negative Conventional PSMA PET/CT Imaging. Diagnostics.

[79] Rosar F., Khreish F., Marlowe R.J., Schaefer-Schuler A., Burgard C., Maus S., Petto S., Bartholoma M., Ezziddin S. (2023). Detection efficacy of [^89^Zr]Zr-PSMA-617 PET/CT in [^68^Ga]Ga-PSMA-11 PET/CT-negative biochemical recurrence of prostate cancer. Eur. J. Nucl. Med. Mol. Imaging.

[80] Rosar F., Schaefer-Schuler A., Bartholoma M., Maus S., Petto S., Burgard C., Prive B.M., Franssen G.M., Derks Y.H.W., Nagarajah J. (2022). [^89^Zr]Zr-PSMA-617 PET/CT in biochemical recurrence of prostate cancer: First clinical experience from a pilot study including biodistribution and dose estimates. Eur. J. Nucl. Med. Mol. Imaging.

[81] van Steenbrugge G.J., Groen M., van Dongen J.W., Bolt J., van der Korput H., Trapman J., Hasenson M., Horoszewicz J. (1989). The human prostatic carcinoma cell line LNCaP and its derivatives. An overview. Urol. Res..

[82] van Steenbrugge G.J., van Uffelen C.J., Bolt J., Schroder F.H. (1991). The human prostatic cancer cell line LNCaP and its derived sublines: An in vitro model for the study of androgen sensitivity. J. Steroid Biochem. Mol. Biol..

[83] Veldscholte J., Berrevoets C.A., Mulder E. (1994). Studies on the human prostatic cancer cell line LNCaP. J. Steroid Biochem. Mol. Biol..

[84] Tai S., Sun Y., Squires J.M., Zhang H., Oh W.K., Liang C.Z., Huang J. (2011). PC3 is a cell line characteristic of prostatic small cell carcinoma. Prostate.

[85] Deri M.A., Zeglis B.M., Francesconi L.C., Lewis J.S. (2013). PET imaging with ^89^Zr: From radiochemistry to the clinic. Nucl. Med. Biol..

[86] Schechter B., Arnon R., Colas C., Burakova T., Wilchek M. (1995). Renal accumulation of streptavidin: Potential use for targeted therapy to the kidney. Kidney Int..

[87] Owens M.C., Zhang C., Liu K.F. (2021). Recent technical advances in the study of nucleic acid modifications. Mol. Cell.

[88] Fantoni N.Z., El-Sagheer A.H., Brown T. (2021). A Hitchhiker’s Guide to Click-Chemistry with Nucleic Acids. Chem. Rev..

[89] Holland J.P., Divilov V., Bander N.H., Smith-Jones P.M., Larson S.M., Lewis J.S. (2010). 89Zr-DFO-J591 for immunoPET of prostate-specific membrane antigen expression in vivo. J. Nucl. Med..

[90] Wen X., Wu Q.P., Ke S., Ellis L., Charnsangavej C., Delpassand A.S., Wallace S., Li C. (2001). Conjugation with (111)In-DTPA-poly(ethylene glycol) improves imaging of anti-EGF receptor antibody C225. J. Nucl. Med..

[91] Wen X., Wu Q.P., Lu Y., Fan Z., Charnsangavej C., Wallace S., Chow D., Li C. (2001). Poly(ethylene glycol)-conjugated anti-EGF receptor antibody C225 with radiometal chelator attached to the termini of polymer chains. Bioconjugate Chem..

[92] Antczak C., Jaggi J.S., LeFave C.V., Curcio M.J., McDevitt M.R., Scheinberg D.A. (2006). Influence of the linker on the biodistribution and catabolism of actinium-225 self-immolative tumor-targeted isotope generators. Bioconjugate Chem..

[93] Li M., Meares C.F. (1993). Synthesis, metal chelate stability studies, and enzyme digestion of a peptide-linked DOTA derivative and its corresponding radiolabeled immunoconjugates. Bioconjugate Chem..

[94] Rosar F., Bartholoma M., Maus S., Prive B.M., Khreish F., Franssen G.M., Derks Y.H.W., Nagarajah J., Ezziddin S. (2022). 89Zr-PSMA-617 PET/CT May Reveal Local Recurrence of Prostate Cancer Unidentified by 68Ga-PSMA-11 PET/CT. Clin. Nucl. Med..

[95] Thevendran R., Tang T.H., Citartan M. (2023). In-silico selection employing rigid docking and molecular dynamic simulation in selecting DNA aptamers against androgen receptor. Biotechnol. J..

[96] Denti V., Monza N., Bindi G., Porto N.S., L’Imperio V., Pagni F., Piga I., Smith A. (2024). 6-Aza-2-Thiothymine as an Alternative Matrix for Spatial Proteomics with MALDI-MSI. Int. J. Mol. Sci..

