# Supplementary material for: Preclinical Evaluation of a Radiolabeled Anti-PSMA Dimeric Aptamer in a Murine Model of Human Prostate Cancer
Source: Molecules. 2026 Jan 31;31(3):493. doi: 10.3390/molecules31030493 (PMC12899641; doi:10.3390/molecules31030493)
Supplement: Supplementary file 1 [file molecules-31-00493-s001.zip › molecules-4094565-supplementary.pdf]

Supplementary Information

Preclinical Evaluation of a Radiolabeled Anti-PSMA Dimeric Aptamer in a Murine Model of Human Prostate Cancer

Akesh Sinha [1], Darpan N. Pandya [1], Prabhakar Eeka [1], Olcay Boyacioglu [2], William H. Gmeiner [3] and Thaddeus J. Wadas [1,*]

[1] Department of Radiology, University of Iowa, Iowa City, IA 52242, USA; akesh-sinha@uiowa.edu (A.S.); darpan-pandya@uiowa.edu (D.N.P.); prabhakar-eeka@uiowa.edu (P.E.)
[2] Department of Food Engineering, Adnan Menderes University, Zafer 09100, Turkey; oboyaci@adu.edu.tr
[3] Department of Cancer Biology, Wake Forest University Health Sciences, Winston-Salem, NC 27109, USA; bgmeiner@wakehealth.edu
* Correspondence: thaddeus-wadas@uiowa.edu; Tel.: +1-319-335-5009

Table of Contents

**Section**	**Page Number**
Figure S1. A 12% native SDS-PAGE gel depicting aptamers A2, A9 and A10.	3
Figure S2. SE-HPLC purification of A10.	4
Figure S3. MALDI-MS spectrum of A11	5
Figure S4. Mass photometry spectrum of A10	6
Figure S5. Mass photometry spectrum of A12	7
Figure S6. Confocal microscopy imaging demonstrates the dimeric aptamer complex binds to PSMA$^+$ LNCAP cells.	8
Figure S7. Biolayer interferometry analysis of the dimeric aptamer complex, A10 for PSMA protein.	9
Figure S8. Size exclusion HPLC purification of A12.	10
Figure S9. Quality control of [^{89}Zr]Zr-A12 using radio-TLC.	11
Figure S10. Quality control of [^{89}Zr]Zr-A12 using radio-HPLC.	12
Figure S11. Stability of [^{89}Zr]Zr-A12 in human serum as assessed by Radio-ITLC.	13
Figure S12. Quality control of [^{89}Zr]Zr-A11 using radio-TLC.	14
Figure S13. Quality control of [^{89}Zr]Zr-A11 using radio-HPLC.	15
Figure S14. Stability of [^{89}Zr]Zr-A11 in human serum at 37°C as assessed by Radio-TLC.	16
Table S1. Complete Biodistribution of [^{89}Zr]Zr-A12.	17
Table S2. Complete Biodistribution of [^{89}Zr]Zr-A11.	18

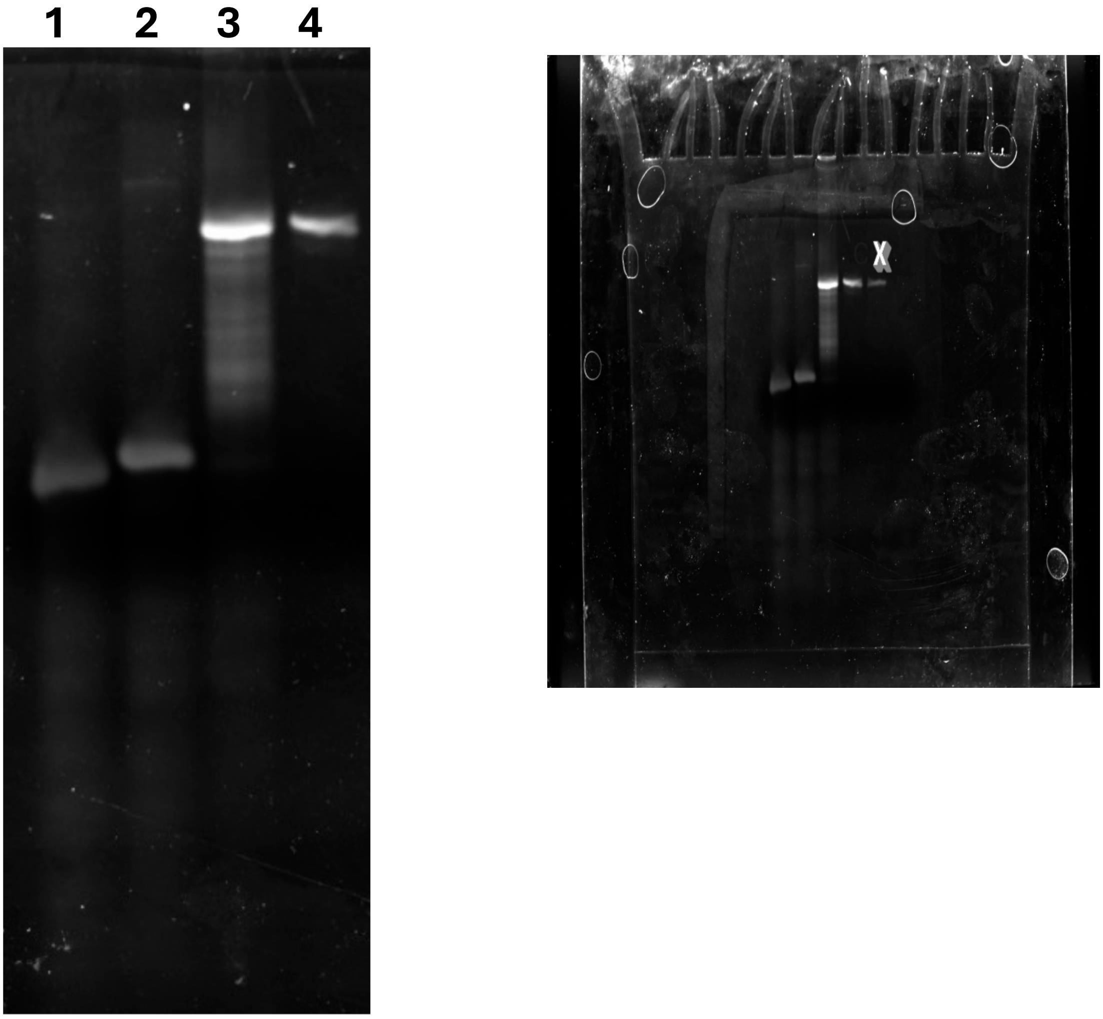

Figure S1. A 12% native SDS-PAGE gel showing different aptamer strands A2, A9 and A10. Left: The gel image shows aptamer strands A2 (Lane 1) and A9 (Lane 2) of the dimeric aptamer complex. The crude, annealed reaction product containing biotinylated dimeric aptamer complex (A10) is depicted in Lane 3. Lane 4 depicts A10 after HPLC purification. **Right:** The original gel. "X" marks the lane that was cropped out of the image on the left and is the triplicate run of lanes 3 and 4.

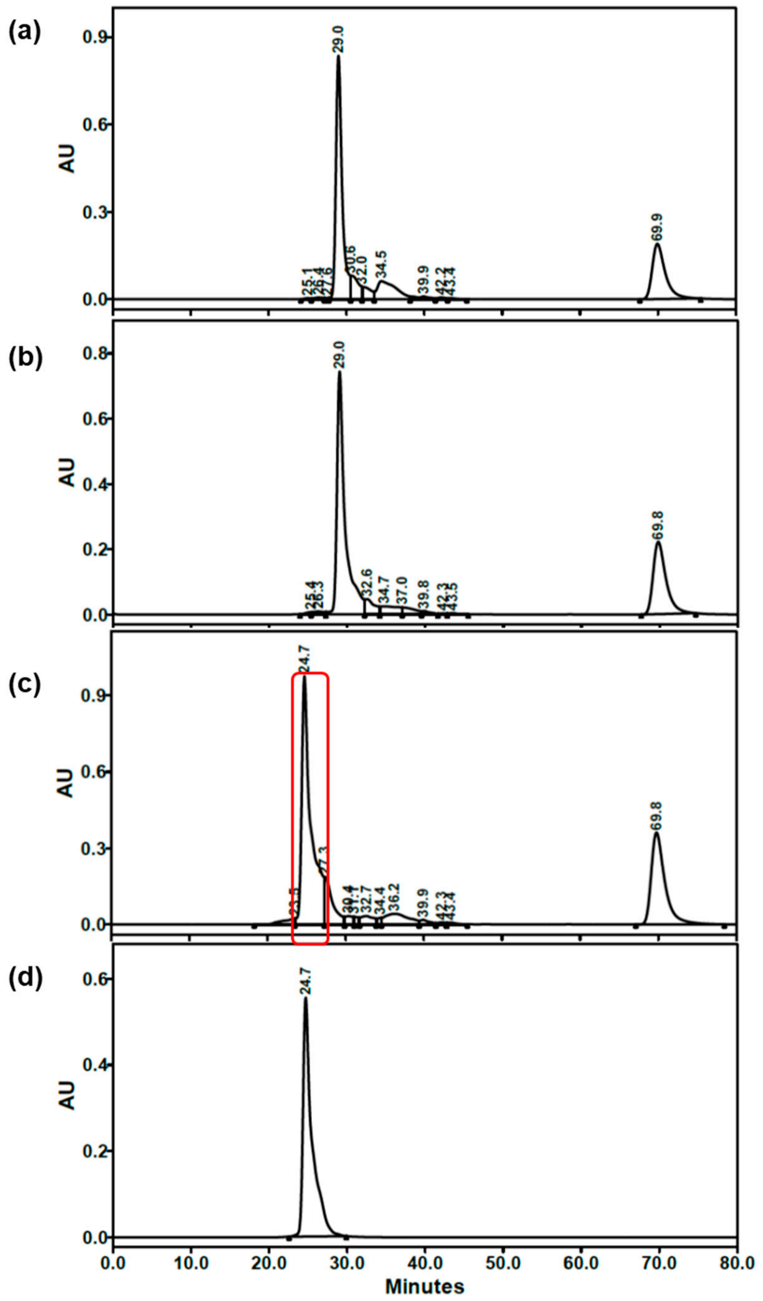

Figure S2. SE-HPLC purification of A10. (a) UV-HPLC chromatogram (220 nm) of monomer A2. (b) biotinylated monomer A9. (c) crude reaction mixture of biotinylated dimer, A10. (d) pure biotinylated dimer, A10 after HPLC purification. The red box denotes the time frame where fractions were collected during purification.

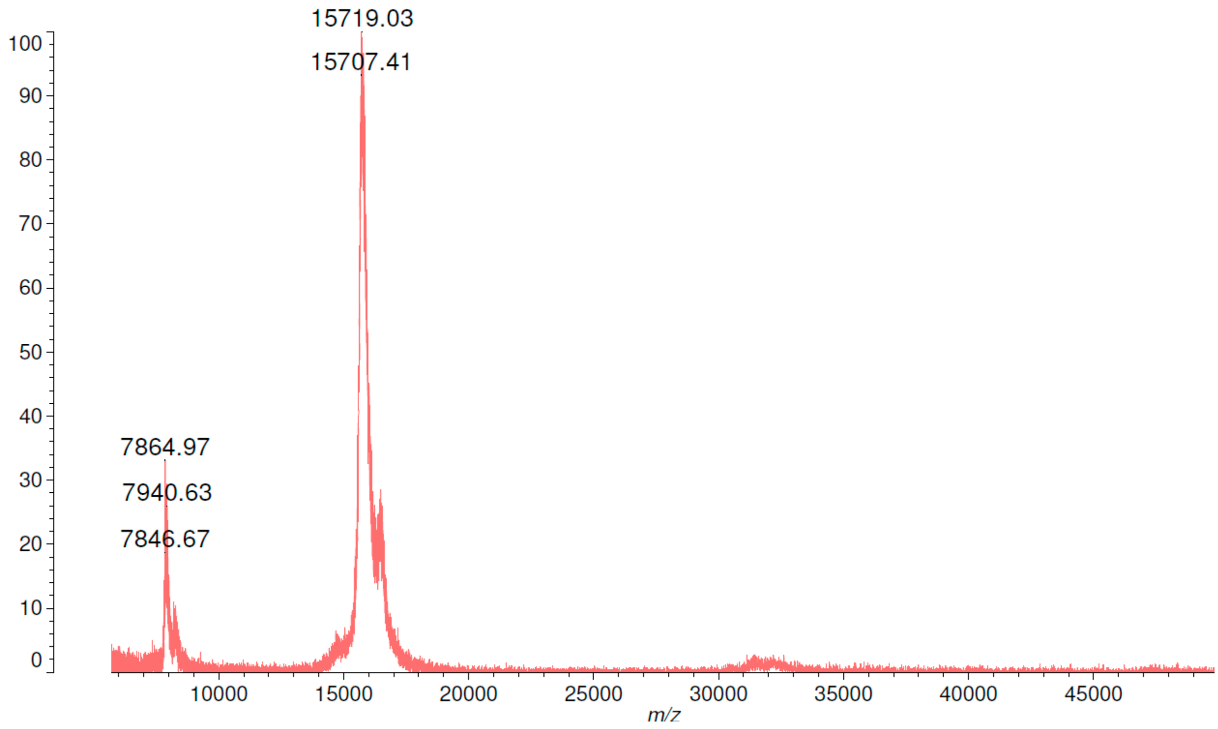

Figure S3. MALDI-MS spectrum of A11. MALDI-MS analysis of the A11 bioconjugate revealed an observed mass of 15.72 kDa. This corresponds to approximately 0.36 Df molecules conjugated to the monomeric SA protein, which has a mass of 15.45 kDa.

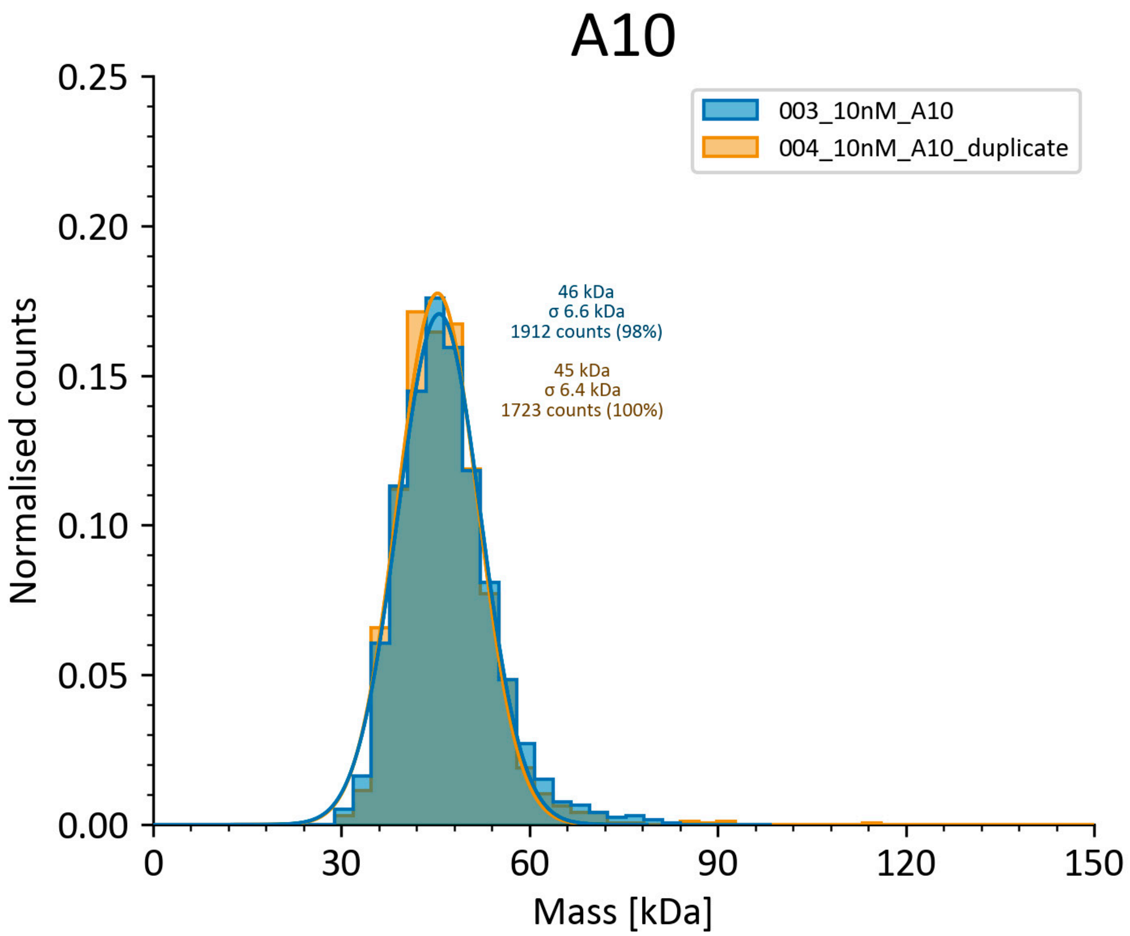

Figure S4. Mass photometry spectrum of A10. MP of A10 revealed a mass of 45.5 ± 6.5 kDa.

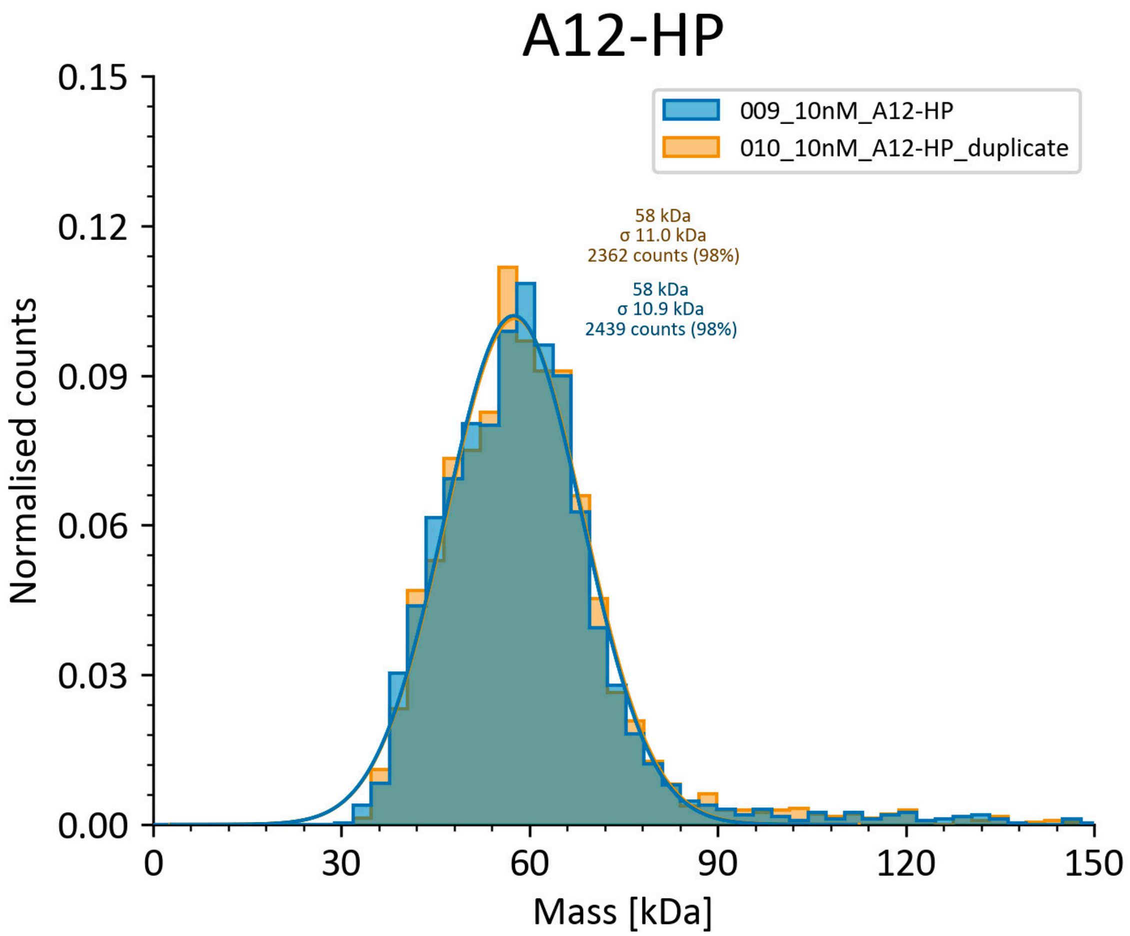

Figure S5. Mass photometry spectrum of A12. MP of the A12 system revealed an average mass of 58 ± 11 kDa.

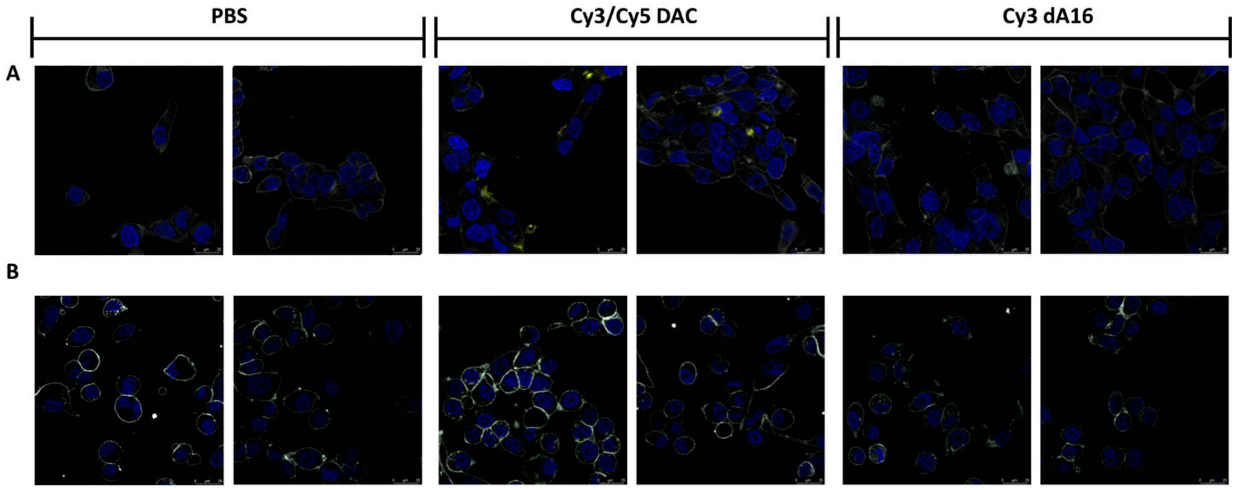

Figure S6. Confocal microscopy imaging demonstrates the dimeric aptamer complex binds to PSMA⁺ PCa cells. Cells were incubated with either D-PBS buffer control or 1 μM of Cy3-Cy5-labeled DAC (A6) or Cy3-labeled dA16 (A7) for 2 hours followed by fixation and imaging. Confocal images are shown for two independent replicates of experiments involving PSMA positive LNCaP cell (A) or PSMA negative PC-3 cells (B). Confocal microscopy shows that Cy3-Cy5-labeled DAC (shown in *Yellow*) was selectively bound and internalized by PSMA positive LNCaP cell line (A) and not by the PSMA negative PC-3 cell line (B). The signal from A6 was absent in LNCaP cells incubated with D-PBS or with A7 oligo. Likewise, no fluorescence was observed in PC-3 cells incubated with any of these reagents. Cell membrane labeled with Alexa 488 labeled wheat germ agglutinin is shown in *Gray* while the DAPI stained nucleus is shown in Blue.

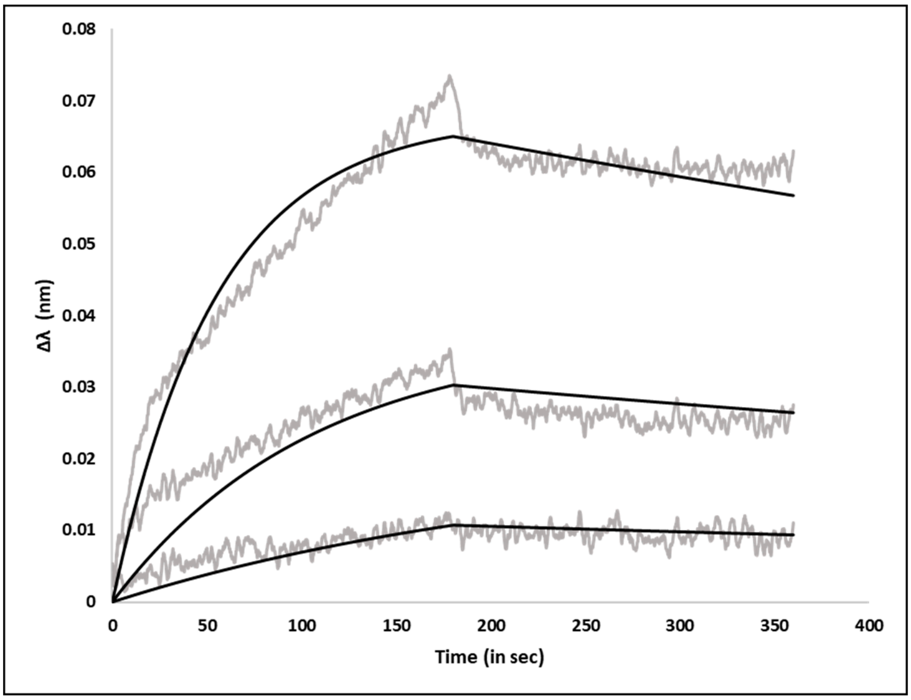

Figure S7. Biolayer interferometry analysis of the dimeric aptamer complex, A10 for PSMA protein. Affinity measurements were conducted using Biolayer interferometry (BLI) in 3 separate binding reactions. The A10 ligand was immobilized to streptavidin sensor in limiting amounts and the dimeric human PSMA analyte was used in varying concentrations (from 0-50 nM) to obtain association and dissociation curves. The 1:1 binding model was used to fit the binding curves.

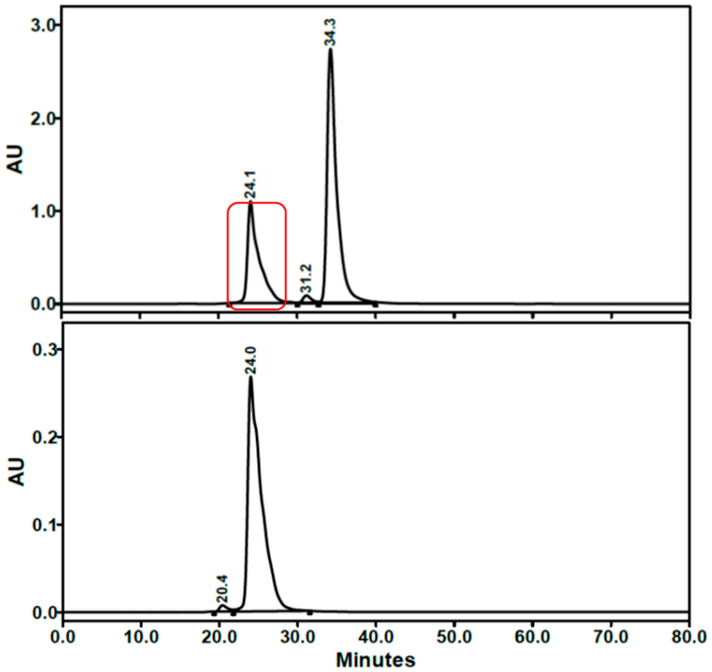

Figure S8. Size exclusion HPLC purification of A12. (upper panel) UV-HPLC chromatogram (220 nm) of crude reaction mixture of A12. **(lower panel)** UV-HPLC chromatogram (220 nm) of purified A12.

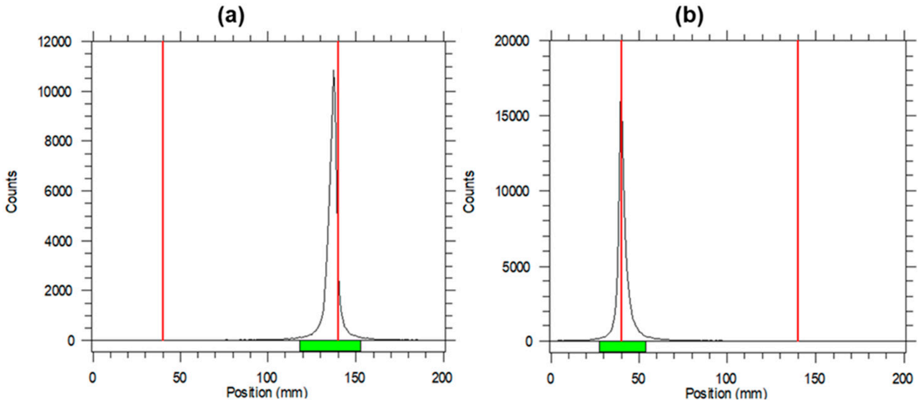

Figure S9. Quality control of [^{89}Zr]Zr-A12 using radio-TLC. (a) Radio-ITLC of [^{89}Zr]Zr(ox)$_4$. **(b)** [^{89}Zr]Zr-A12. In this ITLC-SG system, unchelated [^{89}Zr]Zr(ox)$_4$ was complexed by the eluent EDTA to form [^{89}Zr]Zr-EDTA, which eluted with the solvent front (R_f ~ 1), while [^{89}Zr]Zr-A12 remained at the origin (R_f ~ 0).

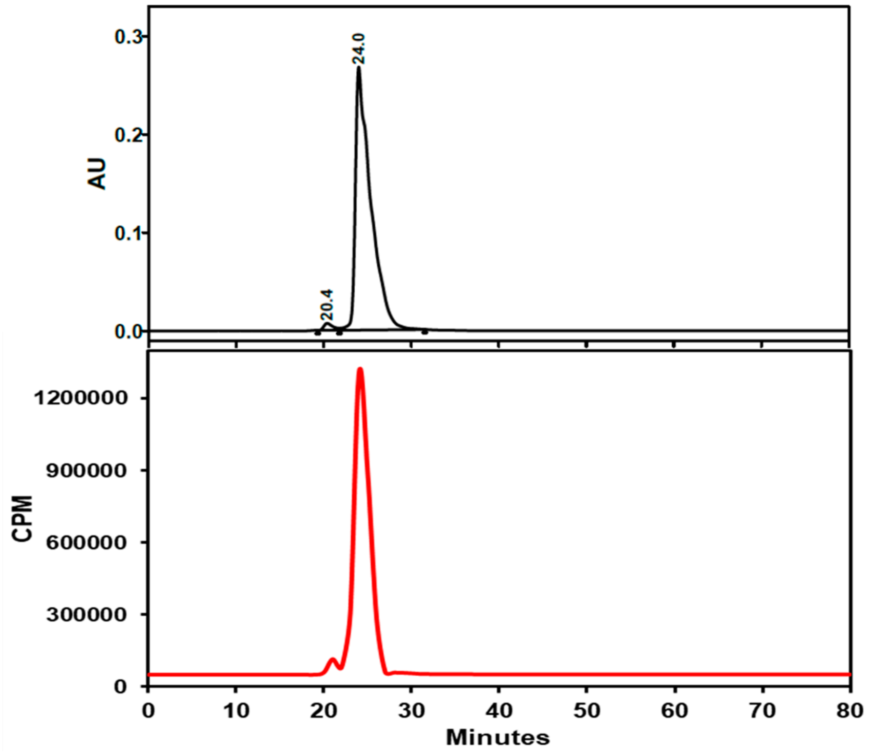

Figure S10. Quality control of [^{89}Zr]Zr-A12 using radio-HPLC. (Upper Panel) UV-HPLC chromatogram (220 nm) of nonradioactive A12. **(Lower Panel)** Radio-HPLC chromatogram of [^{89}Zr]Zr-A12. The retention time of the radiopharmaceutical is similar to that of the non-radioactive dimeric aptamer complex and suggests that [^{89}Zr]Zr-A12 can be prepared with high purity.

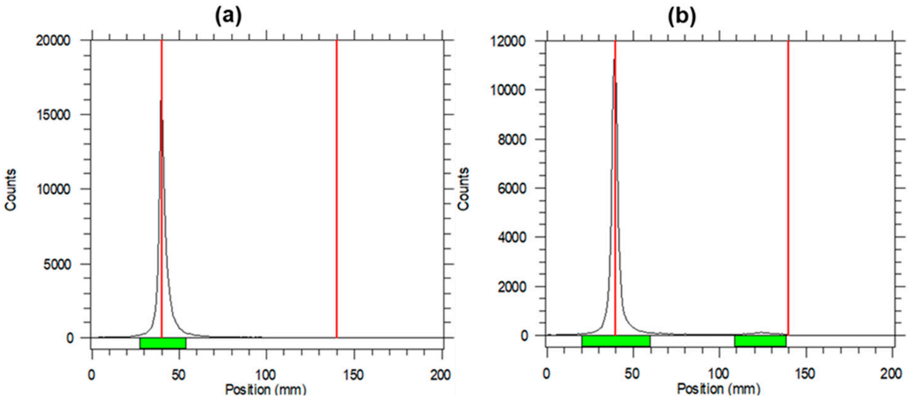

Figure S11. Stability of [^{89}Zr]Zr-A12 in human serum as assessed by radio-ITLC. (a) Stability of [^{89}Zr]Zr-A12 in human serum at 37°C after 0 h. **(b)** Stability of [^{89}Zr]Zr-A12 in human serum at 37°C after 7 days. The mobile phase is 50 mM EDTA (pH 5.0). If transchelation to serum proteins was to occur, then the transchelated ^{89}Zr would form a complex with EDTA and move to the solvent front (R_f ~ 1). Radioactivity was observed to remain at the origin (R_f ~ 0) throughout this study suggesting minimal transchelation of ^{89}Zr from [^{89}Zr]Zr-A12 to serum proteins is observed over the experimental time course.

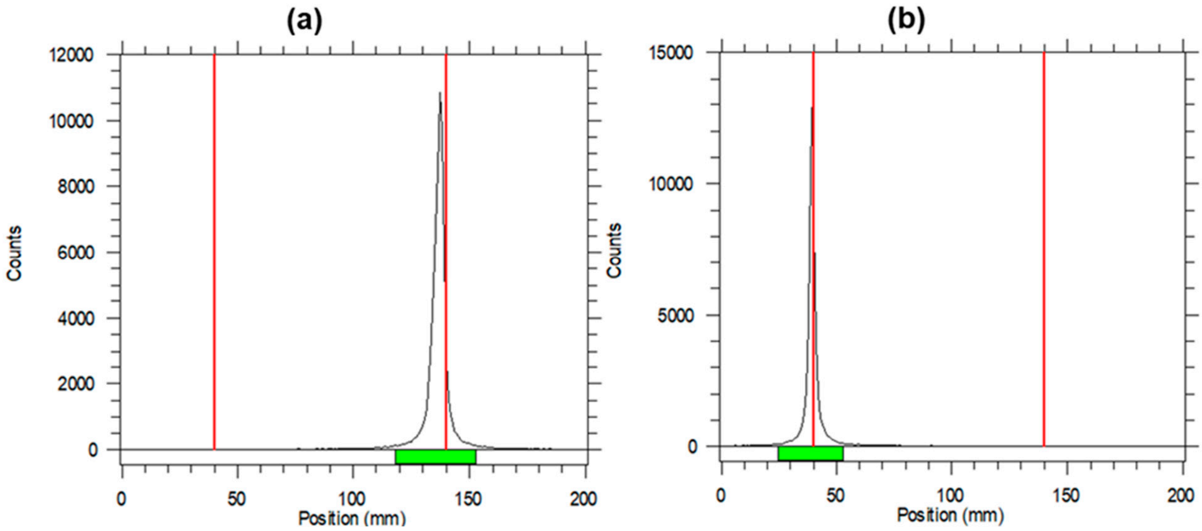

Figure S12. Quality control of [^{89}Zr]Zr-A11 using radio-TLC. (a) Radio-ITLC of [^{89}Zr]Zr(ox)$_4$. **(b)** [^{89}Zr]Zr-A11. In this ITLC-SG system, unchelated [^{89}Zr]Zr(ox)$_4$ was complexed by the eluent EDTA to form [^{89}Zr]Zr-EDTA, which eluted with the solvent front (R_f ~ 1), while [^{89}Zr]Zr-A11 remained at the origin (R_f ~ 0).

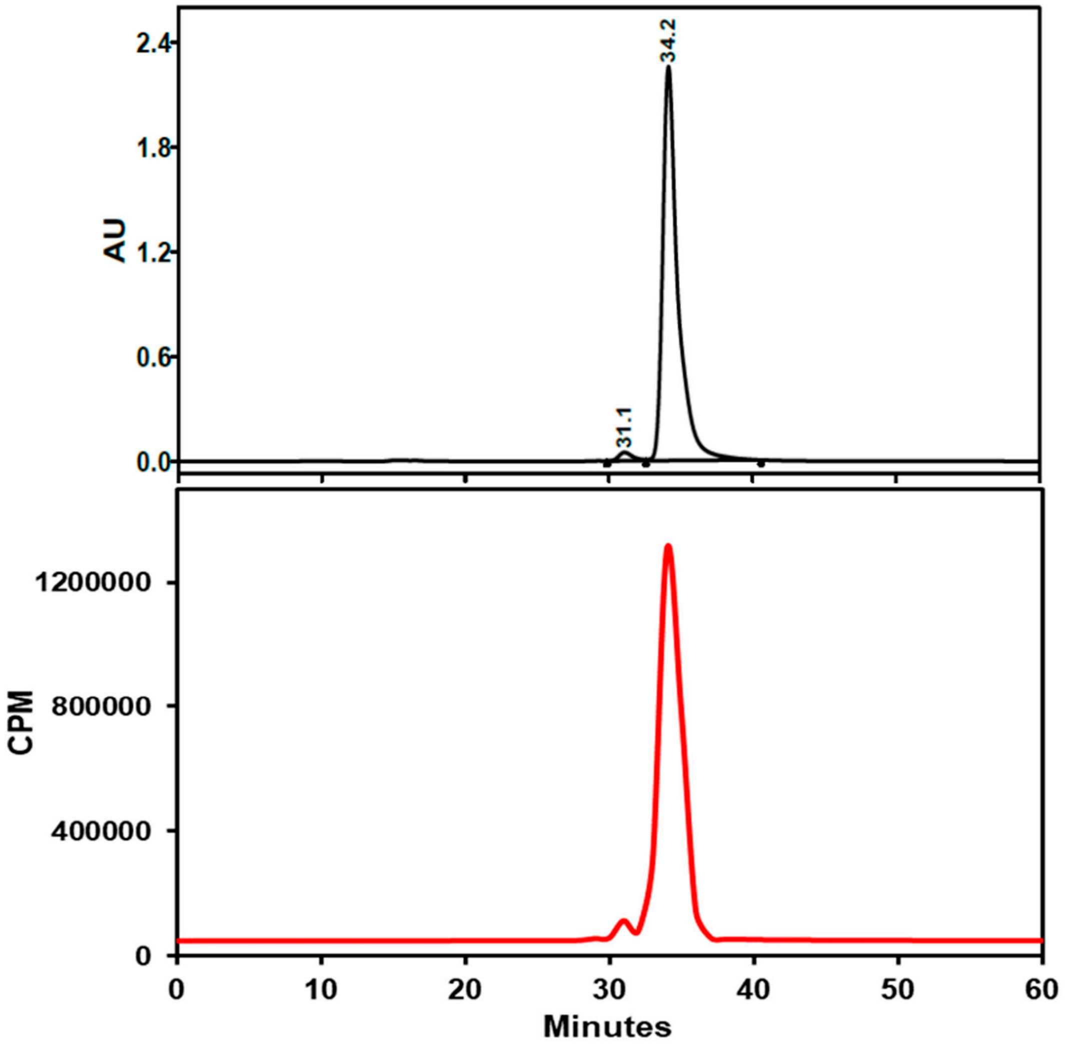

Figure S13. Quality control of [^{89}Zr]Zr-A11 using radio-HPLC. Top Panel: UV-HPLC chromatogram (220 nm) of nonradioactive A11. **Bottom Panel:** Radio-HPLC chromatogram of [^{89}Zr]Zr-A11. Chromatograms demonstrate that [^{89}Zr]Zr-A11 can be prepared with high purity.

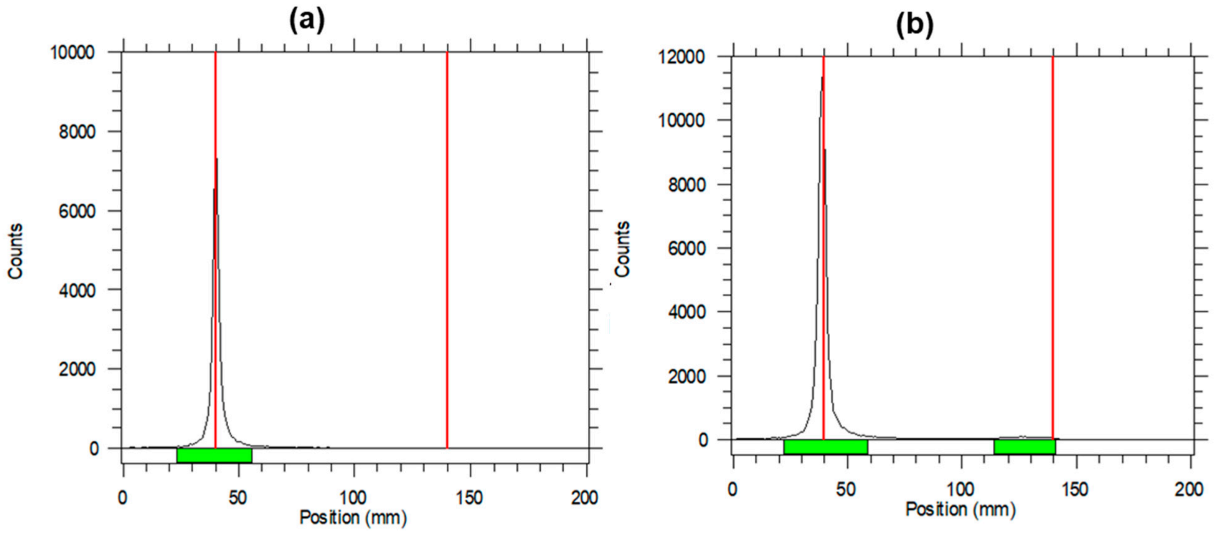

Figure S14. Stability of [^{89}Zr]Zr-A11 in human serum at 37°C as assessed by radio-TLC. (a) Stability of [^{89}Zr]Zr-A11 in human serum at 37°C after 0 h. **(b)** Stability of [^{89}Zr]Zr-A11 in human serum at 37°C after 7 days. The mobile phase is 50 mM EDTA (pH 5.0). If transchelation to serum proteins was to occur, then the transchelated ^{89}Zr would form a complex with EDTA and move to the solvent front (R_f ~ 1). Radioactivity was observed to remain at the origin (R_f ~ 0) throughout this study suggesting minimal transchelation of ^{89}Zr from [^{89}Zr]Zr-A11 to serum proteins is observed over the experimental time course.

Table S1. Complete Biodistribution of [^{89}Zr]Zr-A12.

Tissue/Organ	1 h			4 h			24 h		
Blood	1.242	±	0.274	0.544	±	0.097	0.222	±	0.040
Heart	0.704	±	0.072	0.510	±	0.070	0.400	±	0.050
Lung	1.502	±	0.154	0.972	±	0.127	0.489	±	0.065
Liver	19.763	±	1.375	22.088	±	1.207	21.933	±	2.082
SMI + contents	0.463	±	0.061	0.316	±	0.031	0.253	±	0.034
LGI + contents	0.247	±	0.079	0.668	±	0.086	0.595	±	0.062
Kidney	142.928	±	3.765	140.198	±	9.613	130.222	±	6.265
Spleen	4.160	±	0.468	2.965	±	0.483	3.368	±	0.365
Pancreas	0.237	±	0.023	0.178	±	0.057	0.145	±	0.013
Stomach	0.116	±	0.021	0.071	±	0.017	0.291	±	0.066
Muscle	0.108	±	0.009	0.075	±	0.013	0.040	±	0.015
Fat	0.302	±	0.014	0.253	±	0.075	0.287	±	0.036
Bone	2.542	±	0.141	2.402	±	0.445	3.151	±	0.520
Tumor (+)	0.483	±	0.068	0.339	±	0.035	0.280	±	0.041
Tumor (-)	0.334	±	0.016	0.243	±	0.042	0.195	±	0.034
Tail	0.689	±	0.123	0.965	±	1.255	0.360	±	0.150
Std	1.029	±	0.016	1.016	±	0.014	1.020	±	0.026

Table S2. Complete Biodistribution of [^{89}Zr]Zr-A11.

Tissue/Organ	1 h			4 h			24 h		
Blood	1.011	±	0.102	0.044	±	0.013	0.022	±	0.009
Heart	0.308	±	0.035	0.059	±	0.012	0.047	±	0.015
Lung	0.545	±	0.054	0.265	±	0.030	0.195	±	0.010
Liver	0.395	±	0.029	0.310	±	0.056	0.316	±	0.020
SMI + contents	0.689	±	0.163	0.214	±	0.047	0.100	±	0.017
LGI + contents	0.174	±	0.032	0.786	±	0.084	0.386	±	0.081
Kidney	95.119	±	3.866	151.326	±	14.018	176.565	±	7.777
Spleen	0.245	±	0.028	0.161	±	0.013	0.210	±	0.031
Pancreas	0.159	±	0.023	0.061	±	0.015	0.054	±	0.010
Stomach	0.123	±	0.025	0.077	±	0.022	0.126	±	0.014
Muscle	0.146	±	0.025	0.032	±	0.014	0.014	±	0.010
Fat	0.239	±	0.060	0.057	±	0.020	0.140	±	0.056
Bone	0.190	±	0.032	0.062	±	0.018	0.059	±	0.012
Tumor (+)	0.259	±	0.072	0.144	±	0.038	0.190	±	0.092
Tumor (-)	0.278	±	0.033	0.136	±	0.050	0.129	±	0.031
Tail	27.663	±	4.290	4.421	±	0.560	3.023	±	0.051
Std	0.998	±	0.006	0.989	±	0.014	1.018	±	0.031
